# Crystal structure and DFT study of (*E*)-2-chloro-4-{[2-(2,4-di­nitro­phen­yl)hydrazin-1-yl­idene]meth­yl}phenol aceto­nitrile hemisolvate

**DOI:** 10.1107/S205698901900642X

**Published:** 2019-05-10

**Authors:** Necmi Dege, Md. Serajul Haque Faizi, Onur Erman Doğan, Erbil Ağar, Irina A. Golenya

**Affiliations:** a Ondokuz Mayis University, Faculty of Arts and Sciences, Department of Physics, 55139, Kurupelit, Samsun, Turkey; b Department of Chemistry, Langat Singh College, B. R. A. Bihar University, Muzaffarpur, Bihar 842001, India; c Ondokuz Mayis University, Faculty of Arts and Sciences, Department of Chemistry, 55139, Kurupelit, Samsun, Turkey; d National Taras Shevchenko University, Department of Chemistry, Volodymyrska str., 64, 01601 Kyiv, Ukraine

**Keywords:** crystal structure, hydrazine, 2,4-di­nitro­phen­yl, Schiff base, hydrogen bonding, supra­molecular framework, DFT

## Abstract

The title Schiff base compound, C_13_H_9_ClN_4_O_5_·0.5CH_3_CN, crystallizes as an aceto­nitrile hemisolvate; the solvent mol­ecule being located on a twofold rotation axis. The mol­ecule is nearly planar, with a dihedral angle between the two benzene rings of 3.7 (2)°. The configuration about the C=N bond is *E*, and there is an intra­molecular N—H⋯O_nitro_ hydrogen bond present forming an *S*(6) ring motif. In the crystal, mol­ecules are linked by O—H⋯O and N—H⋯O hydrogen bonds, forming layers lying parallel to (10). The layers are linked by C—H⋯Cl hydrogen bonds, forming a supra­molecular framework. Within the framework there are offset π–π stacking inter­actions [inter­centroid distance = 3.833 (2) Å] present involving inversion-related mol­ecules. The DFT study shows that the HOMO and LUMO are localized in the plane extending from the phenol ring to the 2,4-di­nitro­benzene ring, and the HOMO–LUMO gap is found to be 0.13061 a.u.

## Abstract 2

The title Schiff base compound was obtained from a condensation reaction of 4-chloro-3-hy­droxy­benzaldehyde and 2,4-di­nitro­phenyl­hydrazine. The mol­ecule is almost planar with the dihedral angle between the benzene rings being 3.70 (17)°.

## Chemical context   

Over the past 25 years, extensive research has surrounded the synthesis and use of Schiff base compounds in organic and inorganic chemistry, as they have important medicinal and pharmaceutical applications. These compounds show biological activities including anti­bacterial, anti­fungal, anti­cancer and herbicidal activities (Desai *et al.*, 2001 ▸; Singh & Dash, 1988 ▸; Karia & Parsania, 1999 ▸). Schiff bases are also becoming increasingly important in the dye and plastics industries as well as for liquid-crystal technology and the mechanistic investigation of drugs used in pharmacology, biochemistry and physiology (Sheikhshoaie & Sharif, 2006 ▸). 2,4-Di­nitro­phenyl­hydrazine is frequently used as a reagent for the characterization of aldehydes and ketones (Furniss *et al.*, 1999 ▸). Its derivatives are widely used as dyes (Guillaumont & Nakamura, 2000 ▸). They are also found to have versatile coordinating abilities towards different metal ions (Raj & Kurup, 2007 ▸). The present work is a part of an ongoing structural study of Schiff bases and their utilization in the synthesis of quinoxaline derivatives (Faizi *et al.*, 2016*a* ▸), fluorescence sensors (Faizi *et al.*, 2016*b* ▸) and coordination compounds (Faizi & Prisyazhnaya, 2015 ▸). We report herein on the synthesis, crystal structure and DFT computational calculations of the title new Schiff base compound. The results of calculations by density functional theory (DFT) carried out at the B3LYP/6–311 G(d,p) level are compared with the experimentally determined mol­ecular structure in the solid state.
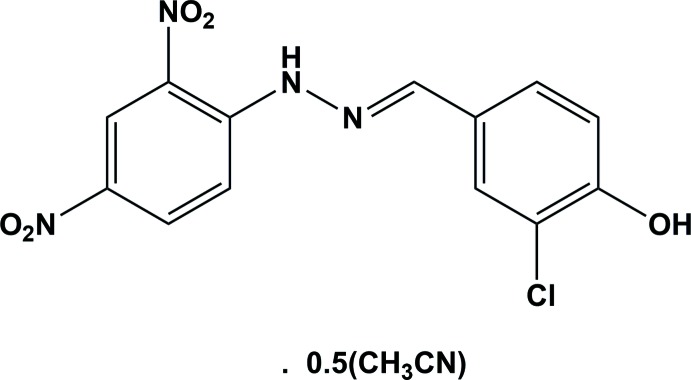


## Structural commentary   

**Figure 1:**
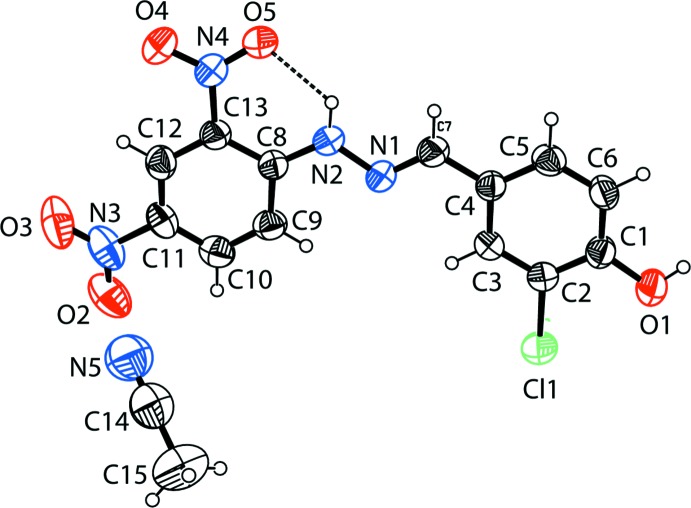
The mol­ecular structure of the title compound, with the atom labelling. Displacement ellipsoids are drawn at the 40% probability level. The intra­molecular N—H⋯O hydrogen bond (see Table 1 ▸), forming an *S*(6) ring motif, is shown as a dashed line.

The mol­ecular structure of the title compound is shown in Fig. 1 ▸. The configuration about the C7=N1 bond is *E*, and there is intra­molecular N—H⋯O_nitro_ hydrogen bond that generates an *S*(6) ring motif (Fig. 1 ▸ and Table 1 ▸). The N1—N2 bond length is 1.380 (3) Å and the N1=C7 bond length is 1.275 (4) Å. These bond lengths are comparable with those of some closely related compounds (Fun *et al.*, 2013 ▸; Faizi *et al.*, 2017 ▸; Ghosh *et al.*, 2016 ▸). The C8—C9 and C8—C13 bonds [1.411 (5) and 1.414 (4) Å, respectively], which are adjacent to the imino N2 atom, are significantly longer than the average distance of 1.375 (3) Å for the other C—C bonds in the same benzene ring. This same pattern of bond lengths has been observed previously in some 2,4-di­nitro­phenyl­hydrazone derivatives (Ohba, 1996 ▸; Borwick *et al.*, 1997 ▸). The title mol­ecule is almost planar with the dihedral angle between the benzene rings being 3.70 (17)°. The nitro groups of the 2,4-di­nitro­phenyl unit are twisted slightly with respect to the C8–C13 benzene ring to which they are attached: nitro group N2/O4/O5 is inclined to the benzene ring by 2.1 (4)°, while nitro group N3/O2/O3 is inclined to it by 6.5 (5)°.

## Supra­molecular features   

**Figure 2:**
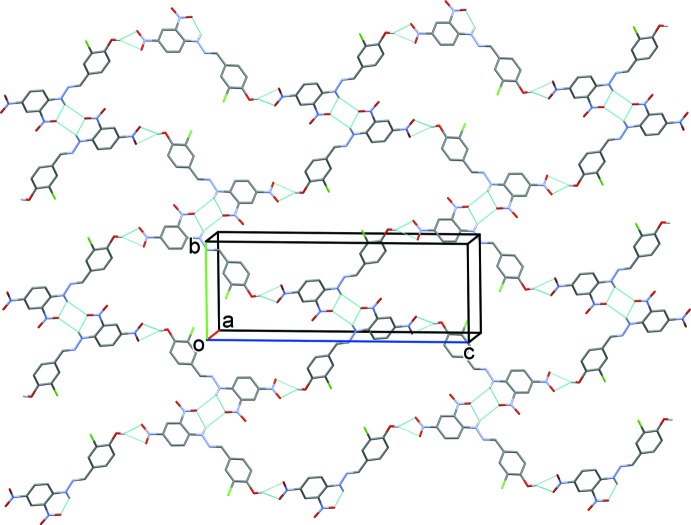
A view along the *a* axis of the crystal packing of the title compound. Hydrogen bonds (see Table 1 ▸) are shown as dashed lines. For clarity, the aceto­nitrile solvent mol­ecules have been omitted and only hydrogen atoms H1 and H2 have been included.

**Figure 3:**
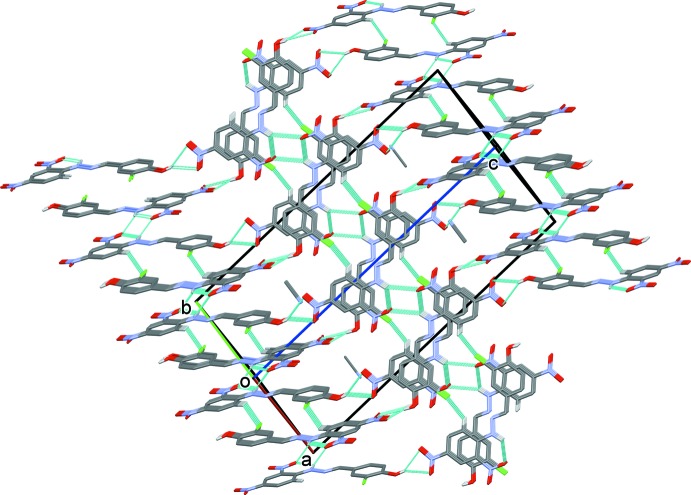
A view normal to plane (110) of the crystal packing of the title compound. Hydrogen bonds (see Table 1 ▸) are shown as dashed lines, and, for clarity, only hydrogen atoms H1, H2 and H9 have been included.

In the crystal, mol­ecules are linked by O—H⋯O and N—H⋯O hydrogen bonds (Table 1 ▸), forming layers lying parallel to (10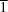), as shown in Fig. 2 ▸. The layers are linked by C—H⋯Cl hydrogen bonds, forming a supra­molecular framework (Fig. 3 ▸ and Table 1 ▸). Within the framework, inversion-related mol­ecules are linked by offset π–π stacking inter­actions (Fig. 3 ▸); *Cg*1⋯*Cg*2^i^ = 3.833 (2) Å, where *Cg*1 and *Cg*2 are the centroids of rings C1–C6 and C8–C13, respectively, α = 3.70 (17)°, β = 27.9°, γ = 24.5°, inter­planar distances are 3.489 (2) and 3.388 (2) Å, offset = 1.791 Å; symmetry code: (i) −*x* + 1, −*y* + 1, −*z* + 1. There are no other significant inter­molecular contacts present in the crystal.

## DFT study   

**Figure 4:**
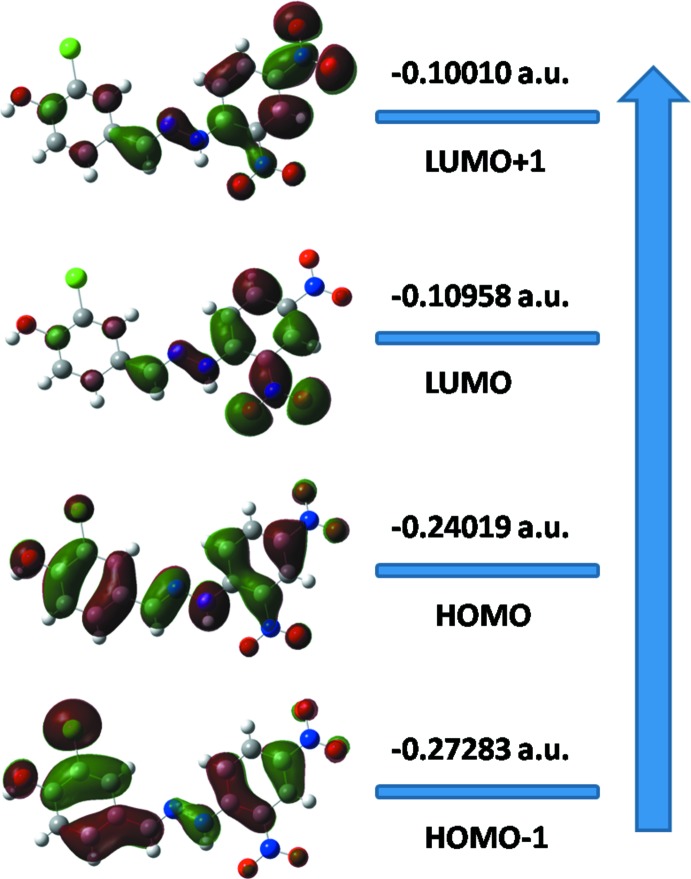
Electron distribution of the HOMO-1, HOMO, LUMO and the LUMO+1 energy levels for the title compound.

The DFT quantum-chemical calculations were performed at the B3LYP/6-311 G(d,p) level (Becke, 1993 ▸) as implemented in *GAUSSIAN09* (Frisch *et al.*, 2009 ▸). The DFT structure optimization of the title compound was performed starting from the X-ray geometry, with experimental values of bond lengths and bond angles matching with theoretical values. The 6-311 G(d,p) basis set is well suited in its approach to the experimental data. The DFT study shows that the HOMO and LUMO are localized in the plane extending from the whole phenol ring to the 2,4-di­nitro­benzene ring. The electron distribution of the HOMO-1, HOMO, LUMO and the LUMO+1 energy levels are shown in Fig. 4 ▸. The HOMO mol­ecular orbital exhibits both σ and π character, whereas HOMO-1 is dominated by π-orbital density. The LUMO is mainly composed of *π*-density while LUMO+1 has both σ and π electronic density. The HOMO–LUMO gap was found to be 0.13061 a.u. and the frontier mol­ecular orbital energies, *E*_HOMO_ and *E*_LUMO_ are −0.24019 and −0.10958 a.u., respectively.

## Database survey   

A search of the Cambridge Structural Database (CSD, version 5.40, update February 2019; Groom *et al.*, 2016 ▸) for the 1-benzyl­idene-2-(2,4-di­nitro­phen­yl)hydrazine skeleton gave 71 hits (see supporting information). 18 of these structures involve a halide substituent and 23 involve a hydroxyl substituent. Only one compound involves both a halide and an hydroxyl substituent and closely resembles the title compound, *viz*. 4-chloro-2-{[(2,4-di­nitro­phen­yl)hydra­zono]meth­yl}phenol (CSD refcode HUTHOV; Ghosh *et al.*, 2016 ▸). Here the benzene rings are inclined to each other by 3.40 (9)°, compared to 3.70 (17)° in the title compound, and again there is an intra­molecular N—H⋯O_nitro_ hydrogen bond present forming an *S*(6) ring motif. In fact, in all 71 structures (see supporting information) there is an intra­molecular N—H⋯O_nitro_ hydrogen bond present forming an *S*(6) ring motif, and in the majority of the compounds the two benzene rings are almost coplanar with the dihedral angle varying between *ca* 0 to 8°, with a few exceptions.

## Synthesis and crystallization   

The title compound was prepared by refluxing a mixture of 4-chloro-3-hy­droxy­benzaldehyde (39.1 mg, 0.25 mmol) in ethanol (15 ml) and 2,4-di­nitro­phenyl­hydrazine (49.5 mg, 0.25 mmol) in ethanol (15 ml). The reaction mixture was stirred for 5 h under reflux. Orange plate-like crystals of the title compound were obtained by slow evaporation of a solution in ethanol (yield 68%, m.p. 542–544K).

## Refinement   

Crystal data, data collection and structure refinement details are summarized in Table 2 ▸. The OH and NH hydrogen atoms and the C-bound H atoms were included in calculated positions and allowed to ride on the parent atoms: O—H = 0.82 Å, N—H = 0.86 Å, C—H = 0.93–0.96 Å with *U*_iso_(H) = 1.5*U*_eq_(O-hydroxyl, C-meth­yl) and 1.2*U*_eq_(N,C) for other H atoms.

## Supplementary Material

Crystal structure: contains datablock(s) I, Global. DOI: 10.1107/S205698901900642X/su5496sup1.cif

Structure factors: contains datablock(s) I. DOI: 10.1107/S205698901900642X/su5496Isup2.hkl

CSD search S1. DOI: 10.1107/S205698901900642X/su5496sup3.pdf

Supporting information file. DOI: 10.1107/S205698901900642X/su5496Isup4.cml

CCDC reference: 1912273

Additional supporting information:  crystallographic information; 3D view; checkCIF report

## Figures and Tables

**Table 1:** Hydrogen-bond geometry (Å, °)

*D*—H⋯*A*	*D*—H	H⋯*A*	*D*⋯*A*	*D*—H⋯*A*
N2—H2⋯O5	0.86	2.01	2.619 (4)	127
O1—H1⋯O2^i^	0.82	2.37	3.114 (4)	152
O1—H1⋯O3^i^	0.82	2.25	2.998 (4)	152
N2—H2⋯O5^ii^	0.86	2.58	3.362 (4)	152
C9—H9⋯Cl1^iii^	0.93	2.72	3.485 (4)	140

Symmetry codes: (i) 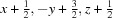; (ii) 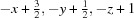; (iii) 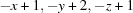.

**Table 2:** Experimental details

Crystal data	
Chemical formula	C_13_H_9_ClN_4_O_5_·0.5C_2_H_3_N
*M*_r_	357.22
Crystal system, space group	Monoclinic, *C*2/*c*
Temperature (K)	296
*a*, *b*, *c* (Å)	12.0614 (11), 9.6960 (6), 26.688 (2)
β (°)	99.619 (7)
*V* (Å^3^)	3077.2 (4)
*Z*	8
Radiation type	Mo *K*α
μ (mm^−1^)	0.29
Crystal size (mm)	0.49 × 0.28 × 0.04

Data collection	
Diffractometer	Stoe IPDS 2
Absorption correction	Integration (*X-RED32*; Stoe & Cie, 2002 ▸)
*T*_min_, *T*_max_	0.908, 0.989
No. of measured, independent and observed [*I* > 2σ(*I*)] reflections	8928, 3027, 1416
*R*_int_	0.056
(sin θ/λ)_max_ (Å^−1^)	0.617

Refinement	
*R*[*F* ^2^ > 2σ(*F* ^2^)], *wR*(*F* ^2^), *S*	0.057, 0.137, 0.93
No. of reflections	3027
No. of parameters	224
H-atom treatment	H-atom parameters constrained
Δρ_max_, Δρ_min_ (e Å^−3^)	0.30, −0.26

Computer programs: *X-AREA* and *X-RED32* (Stoe & Cie, 2002 ▸), *SHELXT2018* (Sheldrick, 2015*a* ▸), *SHELXL2018* (Sheldrick, 2015*b* ▸), *Mercury* (Macrae *et al.*, 2008 ▸), *ORTEP-3 for Windows* and *WinGX* (Farrugia, 2012 ▸) and *PLATON* (Spek, 2009 ▸).

## supplementary crystallographic information

### Crystal data

**Table:**

C_13_H_9_ClN_4_O_5_·0.5C_2_H_3_N	*F*(000) = 1464
*M**_r_* = 357.22	*D*_x_ = 1.542 Mg m^−^^3^
Monoclinic, *C*2/*c*	Mo *K*α radiation, λ = 0.71073 Å
*a* = 12.0614 (11) Å	Cell parameters from 8879 reflections
*b* = 9.6960 (6) Å	θ = 1.6–27.9°
*c* = 26.688 (2) Å	µ = 0.28 mm^−^^1^
β = 99.619 (7)°	*T* = 296 K
*V* = 3077.2 (4) Å^3^	Plate, orange
*Z* = 8	0.49 × 0.28 × 0.04 mm

### Data collection

**Table:**

Stoe IPDS 2 diffractometer	3027 independent reflections
Radiation source: sealed X-ray tube, 12 x 0.4 mm long-fine focus	1416 reflections with *I* > 2σ(*I*)
Plane graphite monochromator	*R*_int_ = 0.056
Detector resolution: 6.67 pixels mm^-1^	θ_max_ = 26.0°, θ_min_ = 1.6°
rotation method scans	*h* = −14→14
Absorption correction: integration (X-RED32; Stoe & Cie, 2002)	*k* = −11→11
*T*_min_ = 0.908, *T*_max_ = 0.989	*l* = −32→32
8928 measured reflections	

### Refinement

**Table:**

Refinement on *F*^2^	Primary atom site location: structure-invariant direct methods
Least-squares matrix: full	Secondary atom site location: difference Fourier map
*R*[*F*^2^ > 2σ(*F*^2^)] = 0.057	Hydrogen site location: mixed
*wR*(*F*^2^) = 0.137	H-atom parameters constrained
*S* = 0.93	*w* = 1/[σ^2^(*F*_o_^2^) + (0.0568*P*)^2^] where *P* = (*F*_o_^2^ + 2*F*_c_^2^)/3
3027 reflections	(Δ/σ)_max_ < 0.001
224 parameters	Δρ_max_ = 0.30 e Å^−^^3^
0 restraints	Δρ_min_ = −0.26 e Å^−^^3^

### Special details

**Table:**

Geometry. All esds (except the esd in the dihedral angle between two l.s. planes) are estimated using the full covariance matrix. The cell esds are taken into account individually in the estimation of esds in distances, angles and torsion angles; correlations between esds in cell parameters are only used when they are defined by crystal symmetry. An approximate (isotropic) treatment of cell esds is used for estimating esds involving l.s. planes.

### Fractional atomic coordinates and isotropic or equivalent isotropic displacement parameters (Å^2^)

**Table:**

	*x*	*y*	*z*	*U*_iso_*/*U*_eq_	Occ. (<1)
Cl1	0.52434 (10)	1.07475 (11)	0.57323 (4)	0.0964 (4)	
O1	0.6912 (2)	1.0735 (3)	0.66391 (9)	0.0832 (8)	
H1	0.733227	1.065785	0.691276	0.125*	
N1	0.6314 (2)	0.5959 (3)	0.49923 (11)	0.0606 (7)	
N2	0.6383 (2)	0.4785 (3)	0.47069 (11)	0.0633 (8)	
H2	0.684398	0.413575	0.481954	0.076*	
O4	0.6517 (2)	0.1472 (3)	0.37327 (11)	0.0926 (9)	
N4	0.6512 (3)	0.2398 (3)	0.40402 (13)	0.0736 (9)	
O5	0.7122 (3)	0.2383 (3)	0.44573 (11)	0.0972 (9)	
O2	0.2825 (3)	0.5344 (4)	0.27290 (12)	0.1162 (12)	
N3	0.3566 (3)	0.4469 (5)	0.28233 (14)	0.0906 (11)	
C4	0.6977 (3)	0.7239 (3)	0.57388 (12)	0.0547 (8)	
O3	0.3675 (3)	0.3539 (4)	0.25304 (12)	0.1189 (12)	
C8	0.5723 (3)	0.4668 (3)	0.42510 (13)	0.0572 (9)	
C7	0.6998 (3)	0.6042 (3)	0.54105 (14)	0.0620 (9)	
H7	0.751146	0.533624	0.550646	0.074*	
C1	0.6946 (3)	0.9554 (3)	0.63640 (13)	0.0622 (9)	
C3	0.6217 (3)	0.8310 (3)	0.55996 (12)	0.0600 (9)	
H3	0.571249	0.825918	0.529585	0.072*	
C2	0.6210 (3)	0.9437 (3)	0.59087 (13)	0.0618 (9)	
C13	0.5754 (3)	0.3556 (3)	0.39104 (13)	0.0582 (9)	
C9	0.4926 (3)	0.5706 (4)	0.40833 (14)	0.0666 (9)	
H9	0.486539	0.645038	0.429653	0.080*	
C5	0.7703 (3)	0.7353 (4)	0.61968 (13)	0.0653 (10)	
H5	0.820955	0.664350	0.629757	0.078*	
C12	0.5062 (3)	0.3507 (4)	0.34458 (13)	0.0664 (10)	
H12	0.510551	0.276937	0.322720	0.080*	
C11	0.4313 (3)	0.4545 (4)	0.33077 (13)	0.0683 (10)	
C6	0.7698 (3)	0.8491 (4)	0.65093 (13)	0.0658 (10)	
H6	0.819628	0.854208	0.681488	0.079*	
C10	0.4249 (3)	0.5663 (4)	0.36269 (15)	0.0724 (10)	
H10	0.374492	0.637587	0.352688	0.087*	
C14	0.500000	0.8470 (10)	0.250000	0.108 (2)	
N5	0.500000	0.7307 (8)	0.250000	0.129 (2)	
C15	0.500000	0.9945 (8)	0.250000	0.133 (3)	
H15A	0.477479	1.027514	0.280695	0.200*	0.5
H15B	0.448298	1.027514	0.221207	0.200*	0.5
H15C	0.574223	1.027514	0.248098	0.200*	0.5

### Atomic displacement parameters (Å^2^)

**Table:**

	*U*^11^	*U*^22^	*U*^33^	*U*^12^	*U*^13^	*U*^23^
Cl1	0.1112 (8)	0.0726 (7)	0.0951 (8)	0.0330 (6)	−0.0125 (6)	−0.0092 (6)
O1	0.096 (2)	0.0762 (17)	0.0716 (18)	0.0121 (15)	−0.0028 (15)	−0.0197 (14)
N1	0.0692 (19)	0.0535 (17)	0.0591 (18)	0.0024 (14)	0.0106 (17)	−0.0066 (14)
N2	0.074 (2)	0.0512 (17)	0.0631 (19)	0.0102 (14)	0.0082 (17)	−0.0038 (14)
O4	0.116 (2)	0.0702 (17)	0.0891 (19)	0.0191 (16)	0.0082 (18)	−0.0220 (16)
N4	0.089 (2)	0.063 (2)	0.068 (2)	0.0086 (18)	0.009 (2)	−0.0067 (18)
O5	0.123 (2)	0.0750 (19)	0.0822 (19)	0.0406 (17)	−0.0170 (18)	−0.0116 (15)
O2	0.086 (2)	0.166 (3)	0.089 (2)	0.016 (2)	−0.0102 (19)	0.023 (2)
N3	0.067 (2)	0.142 (4)	0.063 (2)	−0.009 (3)	0.010 (2)	0.012 (2)
C4	0.058 (2)	0.0523 (19)	0.054 (2)	−0.0033 (17)	0.0103 (18)	0.0007 (17)
O3	0.102 (2)	0.181 (4)	0.068 (2)	0.004 (2)	−0.0016 (18)	−0.029 (2)
C8	0.065 (2)	0.052 (2)	0.054 (2)	−0.0015 (17)	0.0092 (19)	0.0003 (17)
C7	0.067 (2)	0.057 (2)	0.063 (2)	0.0063 (17)	0.013 (2)	−0.0013 (18)
C1	0.067 (2)	0.061 (2)	0.059 (2)	0.0006 (19)	0.0101 (19)	−0.0087 (18)
C3	0.065 (2)	0.058 (2)	0.054 (2)	0.0043 (18)	0.0010 (18)	0.0017 (17)
C2	0.068 (2)	0.055 (2)	0.061 (2)	0.0074 (18)	0.006 (2)	0.0002 (18)
C13	0.060 (2)	0.056 (2)	0.059 (2)	0.0045 (17)	0.0111 (19)	0.0004 (17)
C9	0.070 (2)	0.060 (2)	0.071 (3)	0.009 (2)	0.015 (2)	0.0028 (19)
C5	0.060 (2)	0.071 (2)	0.064 (2)	0.0143 (19)	0.009 (2)	0.003 (2)
C12	0.075 (3)	0.071 (2)	0.055 (2)	−0.005 (2)	0.016 (2)	−0.0022 (19)
C11	0.065 (2)	0.089 (3)	0.051 (2)	−0.007 (2)	0.010 (2)	0.007 (2)
C6	0.064 (2)	0.076 (2)	0.053 (2)	0.003 (2)	−0.0012 (18)	−0.0052 (19)
C10	0.071 (3)	0.075 (2)	0.072 (3)	0.010 (2)	0.013 (2)	0.011 (2)
C14	0.090 (5)	0.123 (7)	0.110 (6)	0.000	0.012 (4)	0.000
N5	0.121 (5)	0.120 (5)	0.137 (5)	0.000	−0.001 (4)	0.000
C15	0.103 (6)	0.103 (6)	0.196 (9)	0.000	0.029 (6)	0.000

### Geometric parameters (Å, º)

**Table:**

Cl1—C2	1.736 (3)	C3—C2	1.370 (4)
O1—C1	1.364 (4)	C3—H3	0.9300
O1—H1	0.8200	C13—C12	1.375 (5)
N1—C7	1.275 (4)	C9—C10	1.349 (5)
N1—N2	1.380 (3)	C9—H9	0.9300
N2—C8	1.343 (4)	C5—C6	1.384 (4)
N2—H2	0.8600	C5—H5	0.9300
O4—N4	1.217 (3)	C12—C11	1.362 (5)
N4—O5	1.228 (4)	C12—H12	0.9300
N4—C13	1.452 (4)	C11—C10	1.388 (5)
O2—N3	1.228 (5)	C6—H6	0.9300
N3—O3	1.215 (4)	C10—H10	0.9300
N3—C11	1.449 (5)	C14—N5	1.128 (9)
C4—C5	1.384 (4)	C14—C15	1.430 (10)
C4—C3	1.394 (4)	C15—H15A	0.9600
C4—C7	1.457 (4)	C15—H15B	0.9600
C8—C9	1.411 (5)	C15—H15C	0.9600
C8—C13	1.414 (4)	C15—H15A^i^	0.9600
C7—H7	0.9300	C15—H15B^i^	0.9600
C1—C2	1.384 (5)	C15—H15C^i^	0.9600
C1—C6	1.385 (5)		
			
C1—O1—H1	109.5	C4—C5—H5	119.0
C7—N1—N2	116.5 (3)	C6—C5—H5	119.0
C8—N2—N1	119.2 (3)	C11—C12—C13	119.6 (3)
C8—N2—H2	120.4	C11—C12—H12	120.2
N1—N2—H2	120.4	C13—C12—H12	120.2
O4—N4—O5	122.1 (3)	C12—C11—C10	120.9 (4)
O4—N4—C13	118.9 (3)	C12—C11—N3	119.3 (4)
O5—N4—C13	118.9 (3)	C10—C11—N3	119.8 (4)
O3—N3—O2	122.3 (4)	C5—C6—C1	119.5 (3)
O3—N3—C11	119.6 (4)	C5—C6—H6	120.2
O2—N3—C11	118.1 (4)	C1—C6—H6	120.2
C5—C4—C3	117.8 (3)	C9—C10—C11	119.4 (4)
C5—C4—C7	121.5 (3)	C9—C10—H10	120.3
C3—C4—C7	120.6 (3)	C11—C10—H10	120.3
N2—C8—C9	119.8 (3)	N5—C14—C15	180.0
N2—C8—C13	124.8 (3)	C14—C15—H15A	109.5
C9—C8—C13	115.4 (3)	C14—C15—H15B	109.5
N1—C7—C4	120.2 (3)	H15A—C15—H15B	109.5
N1—C7—H7	119.9	C14—C15—H15C	109.5
C4—C7—H7	119.9	H15A—C15—H15C	109.5
O1—C1—C2	117.9 (3)	H15B—C15—H15C	109.5
O1—C1—C6	123.4 (3)	C14—C15—H15A^i^	109.471 (2)
C2—C1—C6	118.6 (3)	H15A—C15—H15A^i^	141.1
C2—C3—C4	120.2 (3)	H15B—C15—H15A^i^	56.3
C2—C3—H3	119.9	H15C—C15—H15A^i^	56.2
C4—C3—H3	119.9	C14—C15—H15B^i^	109.470 (3)
C3—C2—C1	121.8 (3)	H15A—C15—H15B^i^	56.3
C3—C2—Cl1	119.5 (3)	H15B—C15—H15B^i^	141.1
C1—C2—Cl1	118.8 (3)	H15C—C15—H15B^i^	56.3
C12—C13—C8	121.9 (3)	H15A^i^—C15—H15B^i^	109.5
C12—C13—N4	116.8 (3)	C14—C15—H15C^i^	109.470 (5)
C8—C13—N4	121.3 (3)	H15A—C15—H15C^i^	56.3
C10—C9—C8	122.8 (4)	H15B—C15—H15C^i^	56.2
C10—C9—H9	118.6	H15C—C15—H15C^i^	141.1
C8—C9—H9	118.6	H15A^i^—C15—H15C^i^	109.5
C4—C5—C6	122.0 (3)	H15B^i^—C15—H15C^i^	109.5
			
C7—N1—N2—C8	−176.8 (3)	O4—N4—C13—C8	−179.3 (3)
N1—N2—C8—C9	−3.5 (4)	O5—N4—C13—C8	1.0 (5)
N1—N2—C8—C13	176.4 (3)	N2—C8—C9—C10	178.9 (3)
N2—N1—C7—C4	−179.4 (3)	C13—C8—C9—C10	−1.0 (5)
C5—C4—C7—N1	179.5 (3)	C3—C4—C5—C6	−0.8 (5)
C3—C4—C7—N1	−0.4 (5)	C7—C4—C5—C6	179.4 (3)
C5—C4—C3—C2	0.7 (5)	C8—C13—C12—C11	−0.7 (5)
C7—C4—C3—C2	−179.4 (3)	N4—C13—C12—C11	178.3 (3)
C4—C3—C2—C1	0.0 (5)	C13—C12—C11—C10	0.9 (5)
C4—C3—C2—Cl1	−179.1 (2)	C13—C12—C11—N3	−178.4 (3)
O1—C1—C2—C3	177.5 (3)	O3—N3—C11—C12	−5.6 (5)
C6—C1—C2—C3	−0.6 (5)	O2—N3—C11—C12	172.9 (3)
O1—C1—C2—Cl1	−3.4 (4)	O3—N3—C11—C10	175.1 (4)
C6—C1—C2—Cl1	178.5 (3)	O2—N3—C11—C10	−6.4 (5)
N2—C8—C13—C12	−179.2 (3)	C4—C5—C6—C1	0.1 (5)
C9—C8—C13—C12	0.7 (5)	O1—C1—C6—C5	−177.5 (3)
N2—C8—C13—N4	1.8 (5)	C2—C1—C6—C5	0.6 (5)
C9—C8—C13—N4	−178.3 (3)	C8—C9—C10—C11	1.3 (5)
O4—N4—C13—C12	1.7 (5)	C12—C11—C10—C9	−1.2 (5)
O5—N4—C13—C12	−178.0 (3)	N3—C11—C10—C9	178.1 (3)

Symmetry code: (i) −*x*+1, *y*, −*z*+1/2.

### Hydrogen-bond geometry (Å, º)

**Table:**

*D*—H···*A*	*D*—H	H···*A*	*D*···*A*	*D*—H···*A*
N2—H2···O5	0.86	2.01	2.619 (4)	127
O1—H1···O2^ii^	0.82	2.37	3.114 (4)	152
O1—H1···O3^ii^	0.82	2.25	2.998 (4)	152
N2—H2···O5^iii^	0.86	2.58	3.362 (4)	152
C9—H9···Cl1^iv^	0.93	2.72	3.485 (4)	140

Symmetry codes: (ii) *x*+1/2, −*y*+3/2, *z*+1/2; (iii) −*x*+3/2, −*y*+1/2, −*z*+1; (iv) −*x*+1, −*y*+2, −*z*+1.
