# Supplementary material for: Crystal structure and DFT study of (E)-2-chloro-4-{[2-(2,4-di­nitro­phen­yl)hydrazin-1-yl­idene]meth­yl}phenol aceto­nitrile hemisolvate
Source: Acta Crystallogr E Crystallogr Commun. 2019 May 10;75(Pt 6):770–3. doi: 10.1107/S205698901900642X (PMC6658971; doi:10.1107/S205698901900642X)

Search Overview

Search:	search1
Date/Time done:	Mon May 06 11:18:27 2019
Database(s):	CSD version 5.40 updates (Feb 2019)<br>CSD version 5.40 (November 2018)
Restriction Info:	No refcode restrictions applied
Filters:	None
Percentage Completed:	100%
Number of Hits:	71

Single query used. Search found structures that:

match

Query 1

Query 1

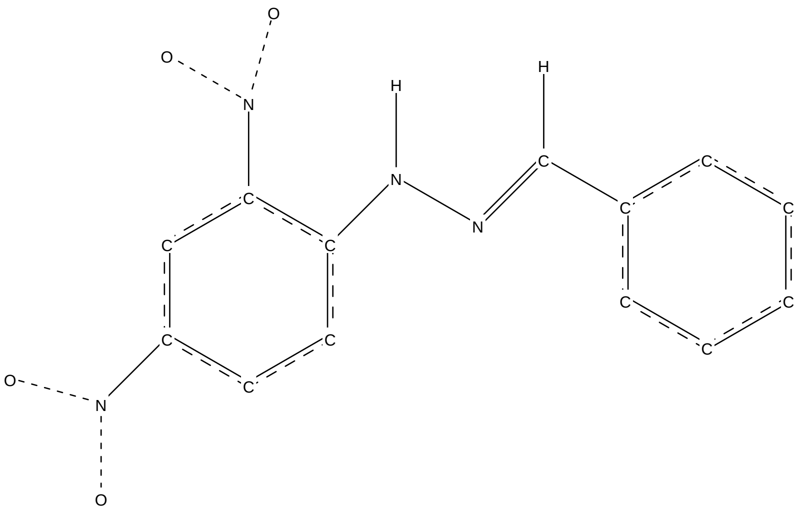

Search: search1 (Mon May 06 11:18:27 2019): Hits 1-4

DILWUT

Reference: T.Sachdeva, S.Bishnoi, M.D.Milton (2017) *Chem. Sel.* ,**2,** 11307

Formula: $C_{27}\ H_{21}\ N_5\ O_4\ S_1$

Compound Name: 3-{[2-(2,4-dinitrophenyl)hydrazinylidene]methyl}-10-ethyl-7-phenyl-10H-phenothiazine

Space Group:	P-1	***Cell:***	***a***	7.807(2)	***b*** 11.146(4)	***c*** 14.314(4)
Space Group No.:	2	***(Å,°)***	α	93.92(3)	β 100.45(2)	γ 100.77(3)

R-Factor (%): 8.81 ***Temperature(K):*** 298 ***Density(g/cm^3):*** 1.420

Parameters

Fragment 1

**ANG1 (A)**	11.226
**DIST1 (D)**	1.994

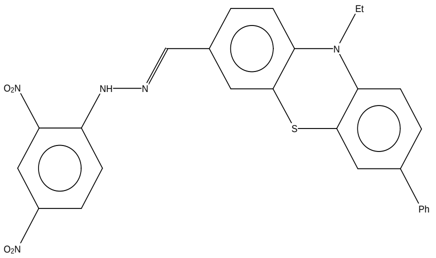

ACEMUR

Reference: J.-L.Wang, Y.-J.Jia (2004) *Pol.J.Chem.* ,**78,**869

Formula: $C_{14}\ H_{10}\ N_4\ O_6$

Compound Name: 3,4-(Methylenedioxy)benzaldehyde 2,4-dinitrophenylhydrazone

Space Group:	P21/c	***Cell:***	***a***	6.606(1)	***b*** 14.354(4)	***c*** 14.549(4)
Space Group No.:	14	***(Å,°)***	α	90.00	β 95.25(0)	γ 90.00

R-Factor (%): 4.94 ***Temperature(K):*** 293 ***Density(g/cm^3):*** 1.597

Parameters

Fragment 1

**ANG1 (A)**	0.772
**DIST1 (D)**	2.040

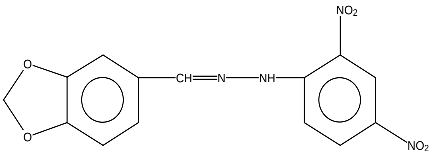

ADENON

Reference: Wen-Zhong Ge, Feng Li (2006) *Acta Crystallogr.,Sect.E:Struct.Rep.Online* ,**62,**o2888

Formula: $C_{13}\ H_8\ Cl_2\ N_4\ O_4$

Compound Name: 2,6-dichlorobenzaldehyde 2,4-dinitrophenylhydrazone

Space Group:	P21/c	***Cell:***	***a***	12.375(2)	***b*** 7.928(1)	***c*** 14.977(2)
Space Group No.:	14	***(Å,°)***	α	90.00	β 101.59(0)	γ 90.00

R-Factor (%): 3.32 ***Temperature(K):*** 298 ***Density(g/cm^3):*** 1.639

Parameters

Fragment 1

**ANG1 (A)**	3.385
**DIST1 (D)**	2.007

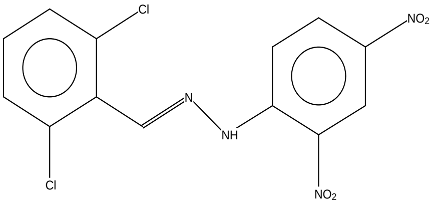

ADEXIR

Reference: Yun Yang, Shu-Lin Ma, Lan-Li Lu, Xiao-Qing Yang (2006) *Acta Crystallogr.,Sect.E:Struct.Rep.Online* ,**62,**o3064

Formula: $C_{16}\ H_{15}\ Br_1\ N_4\ O_6, C_3\ H_7\ N_1\ O_1$

Compound Name: (E)-1-(5-Bromo-2-ethoxy-3-methoxybenzylidene)-2-(2,4-dinitrophenyl) hydrazine dimethylformamide solvate

Space Group:	P-1	***Cell:***	***a***	8.521(2)	***b*** 11.698(3)	***c*** 13.135(4)
Space Group No.:	2	***(Å,°)***	α	64.89(0)	β 73.48(0)	γ 80.84(0)

R-Factor (%): 4.00 ***Temperature(K):*** 294 ***Density(g/cm^3):*** 1.498

Parameters

Fragment 1

**ANG1 (A)**	5.317
**DIST1 (D)**	2.051

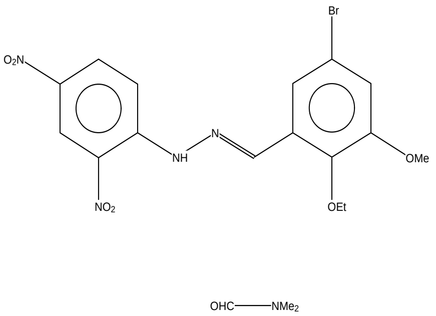

Search: search1 (Mon May 06 11:18:27 2019): Hits 5-8

AFUSEB

Reference: H.-K.Fun, S.Chantrapromma, B.Nilwanna, T.Kobkeatthawin, N.Boonnak (2013) *Acta Crystallogr.,Sect.E:Struct.Rep.Online* ,**69,**o1203

Formula: $C_{16} H_{16} N_4 O_7$

Compound Name: 1-(2,4-Dinitrophenyl)-2-[(E)-2,4,5-trimethoxybenzylidene]hydrazine

Space Group:	P21/c	***Cell:***	***a*** 8.027(1)	***b*** 15.048(2)	***c*** 13.686(2)		
Space Group No.:	14	***(Å,°)***	α 90.00	β 101.55(0)	γ 90.00		
R-Factor (%):	5.51	***Temperature(K):***	100	***Density(g/cm³):***	1.543		

Parameters

Fragment 1
ANG1 (A) 3.146
DIST1 (D) 2.040

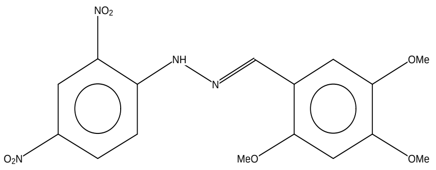

BAFGUL

Reference: B.Szczesna, Z.Urbanczyk-Lipkowska (2002) *New J.Chem.* ,**26,**243

Formula: $C_{13} H_{10} N_4 O_5$

Compound Name: 2-((2,4-Dinitrophenyl)hydrazonomethyl)phenol

Space Group:	P21/n	***Cell:***	***a*** 13.767(0)	***b*** 4.353(0)	***c*** 21.118(0)
Space Group No.:	14	***(Å,°)***	α 90.00	β 90.41(0)	γ 90.00
R-Factor (%):	4.78	***Temperature(K):***	200	***Density(g/cm³):***	1.586

Parameters

Fragment 1
ANG1 (A) 3.793
DIST1 (D) 3.852

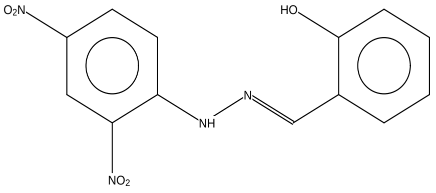

BAFGUL01

Reference: H.H.Monfared, O.Pouralimardan, C.Janiak (2007) *Z.Naturforsch.,B:Chem.Sci.* ,**62,**717

Formula: $C_{13} H_{10} N_4 O_5$

Compound Name: Salicylaldehyde 2,4-dinitrophenylhydrazone

Space Group:	P21/c	***Cell:***	***a*** 13.820(3)	***b*** 4.352(0)	***c*** 25.159(7)
Space Group No.:	14	***(Å,°)***	α 90.00	β 123.01(2)	γ 90.00
R-Factor (%):	4.56	***Temperature(K):***	293	***Density(g/cm³):***	1.582

Parameters

Fragment 1
ANG1 (A) 3.490
DIST1 (D) 3.814

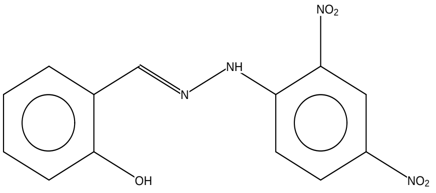

BAFHAS

Reference: B.Szczesna, Z.Urbanczyk-Lipkowska (2002) *New J.Chem.* ,**26,**243

Formula: $C_{13} H_{10} N_4 O_5$

Compound Name: 3-((2,4-Dinitrophenyl)hydrazonomethyl)phenol

Space Group:	P21/n	***Cell:***	***a*** 9.790(1)	***b*** 14.035(1)	***c*** 9.823(1)
Space Group No.:	14	***(Å,°)***	α 90.00	β 101.32(3)	γ 90.00
R-Factor (%):	5.43	***Temperature(K):***	293	***Density(g/cm³):***	1.517

Parameters

Fragment 1
ANG1 (A) 7.783
DIST1 (D) 1.939

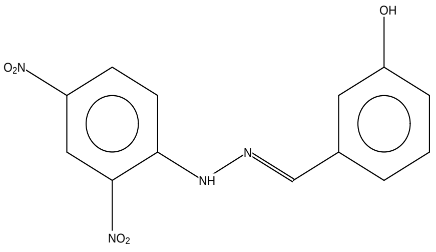

Search: search1 (Mon May 06 11:18:27 2019): Hits 9-12

BAFHEW

Reference: B.Szczesna, Z.Urbanczyk-Lipkowska (2002)
New J.Chem. ,**26,**243

Formula: $C_{13}\ H_{10}\ N_4\ O_5$

Compound Name: 4-((2,4-Dinitrophenyl)hydrazonomethyl)phenol

Space Group:	P21/n	***Cell:***	***a***	8.430(0)	***b***	6.186(0)	***c***	24.715(1)
Space Group No.:	14	***(Å,°)***	α	90.00	β	91.49(1)	γ	90.00

R-Factor (%): 5.71 ***Temperature(K):*** 293 ***Density(g/cm^3):*** 1.558

Parameters

Fragment 1

**ANG1 (A)**	1.914
**DIST1 (D)**	1.901

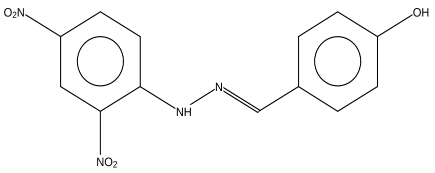

BAFHIA

Reference: B.Szczesna, Z.Urbanczyk-Lipkowska (2002)
New J.Chem. ,**26,**243

Formula: $C_{13}\ H_{10}\ N_4\ O_5,0.5(C_2\ H_3\ N_1)$

Compound Name: 4-((2,4-Dinitrophenyl)hydrazonomethyl)-phenol acetonitrile solvate

Space Group:	C2/c	***Cell:***	***a***	12.002(2)	***b***	9.175(2)	***c***	27.266(5)
Space Group No.:	15	***(Å,°)***	α	90.00	β	102.15(3)	γ	90.00

R-Factor (%): 4.67 ***Temperature(K):*** 293 ***Density(g/cm^3):*** 1.461

Parameters

Fragment 1

**ANG1 (A)**	2.051
**DIST1 (D)**	1.990

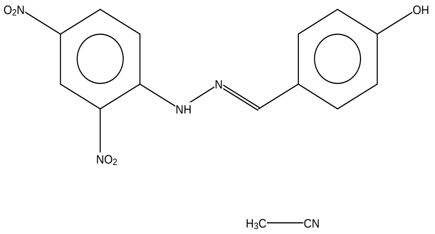

BUWXUO

Reference: M.Kumaravel, J.T.Mague, M.S.Balakrishna (2015)
CSD Communication(Private Communication) ,

Formula: $C_{25}\ H_{19}\ N_4\ O_4\ P_1,C_1\ H_1\ Cl_3$

Compound Name: (E)-1-(2,4-dinitrophenyl)-2-(2-(diphenylphosphino)benzylidene)hydrazine chloroform solvate

Space Group:	P21/n	***Cell:***	***a***	8.019(0)	***b***	11.068(0)	***c***	29.412(0)
Space Group No.:	14	***(Å,°)***	α	90.00	β	90.80(0)	γ	90.00

R-Factor (%): 3.90 ***Temperature(K):*** 150 ***Density(g/cm^3):*** 1.501

Parameters

Fragment 1

**ANG1 (A)**	15.084
**DIST1 (D)**	1.971

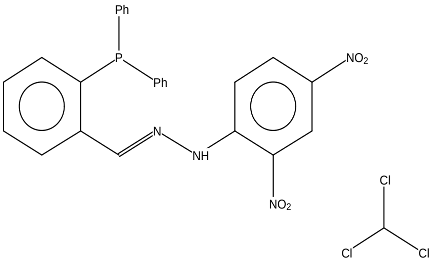

BUWYAV

Reference: M.Kumaravel, J.T.Mague, M.S.Balakrishna (2015)
CSD Communication(Private Communication) ,

Formula: $C_{25}\ H_{19}\ N_4\ O_5\ P_1,H_2\ O_1$

Compound Name: 1-(2,4-dinitrophenyl)-2-(2-(diphenylphosphoryl)benzylidene)hydrazine monohydrate

Space Group:	P-1	***Cell:***	***a***	8.646(0)	***b***	10.765(0)	***c***	12.857(0)
Space Group No.:	2	***(Å,°)***	α	100.74(0)	β	93.50(0)	γ	103.16(0)

R-Factor (%): 3.79 ***Temperature(K):*** 150 ***Density(g/cm^3):*** 1.472

Parameters

Fragment 1

**ANG1 (A)**	4.676
**DIST1 (D)**	1.994

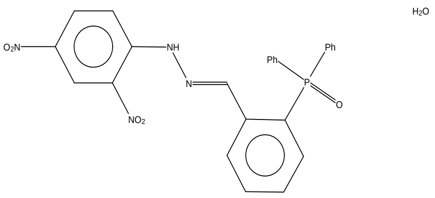

Search: search1 (Mon May 06 11:18:27 2019): Hits 13-16

DADQIJ

Reference: Jin-Ling Wang, Yong-Jin Jia, Fang-Ming Miao, Ai-Xiu Li (2004) *Youji Huaxue(Chin.)(Chin.J.Org.Chem.)* ,**24,**46

Formula: C_{15} H_{15} N_5 O_4

Compound Name: 4-(Dimethylamino)benzaldehyde (2,4-dinitrophenyl)hydrazone

Space Group:	C2/c	***Cell:***	***a***	11.243(6)	***b***	10.526(5)	***c***	26.029(9)
Space Group No.:	15	***(Å,°)***	α	90.00	β	96.22(0)	γ	90.00
R-Factor (%):	5.84	***Temperature(K):***	293	***Density(g/cm³):***	1.429			

Parameters

Fragment 1
ANG1 (A) 1.215
DIST1 (D) 1.991

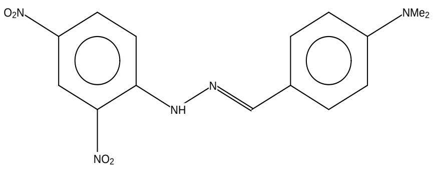

DAYSIG

Reference: Jun Shi (2005) *Acta Crystallogr.,Sect.E:Struct.Rep.Online* , **61,**o4018

Formula: C_{20} H_{16} N_4 O_5

Compound Name: (E)-1-(3-(Benzyloxy)benzylidene)-2-(2,4-dinitrophenyl)hydrazine

Space Group:	P-1	***Cell:***	***a***	5.929(1)	***b***	7.651(2)	***c***	20.087(6)
Space Group No.:	2	***(Å,°)***	α	96.71(0)	β	93.77(0)	γ	90.11(0)
R-Factor (%):	4.24	***Temperature(K):***	294	***Density(g/cm³):***	1.443			

Parameters

Fragment 1
ANG1 (A) 5.033
DIST1 (D) 2.011

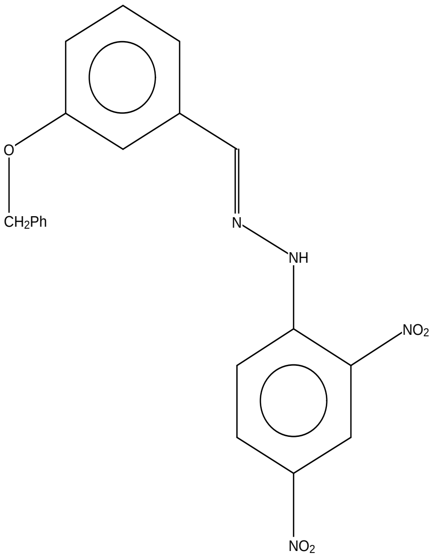

DAYSOM

Reference: Jun Shi (2005) *Acta Crystallogr.,Sect.E:Struct.Rep.Online* , **61,**o4020

Formula: C_{21} H_{18} N_4 O_6,C_2 H_3 N_1

Compound Name: (E)-1-(3-(Benzyloxy)-4-methoxybenzylidene)-2-(2,4-dinitrophenyl) hydrazine acetonitrile solvate

Space Group:	P-1	***Cell:***	***a***	7.945(3)	***b***	12.253(5)	***c***	12.549(5)
Space Group No.:	2	***(Å,°)***	α	67.62(0)	β	84.79(0)	γ	87.73(0)
R-Factor (%):	5.09	***Temperature(K):***	293	***Density(g/cm³):***	1.368			

Parameters

Fragment 1
ANG1 (A) 2.953
DIST1 (D) 2.018

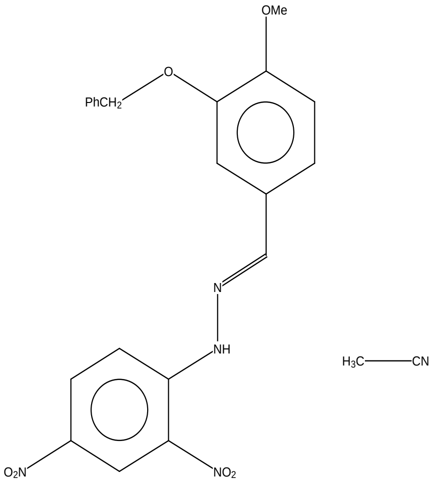

DAYWIK

Reference: Chun-Hua Diao, Zhi Fan, Ming Yu, Xin Chen, Zuo-Liang Jing (2005) *Acta Crystallogr.,Sect.E:Struct.Rep.Online* ,**61,** o4177

Formula: C_{21} H_{17} N_5 O_8

Compound Name: (E)-1-(2,4-Dinitrophenyl)-2-(2-(2-(2-nitrophenoxy)ethoxy)benzylidene) hydrazine

Space Group:	P-1	***Cell:***	***a***	7.953(3)	***b***	11.686(5)	***c***	12.524(5)
Space Group No.:	2	***(Å,°)***	α	65.11(0)	β	87.46(0)	γ	86.98(0)
R-Factor (%):	5.05	***Temperature(K):***	294	***Density(g/cm³):***	1.473			

Parameters

Fragment 1
ANG1 (A) 2.921
DIST1 (D) 1.989

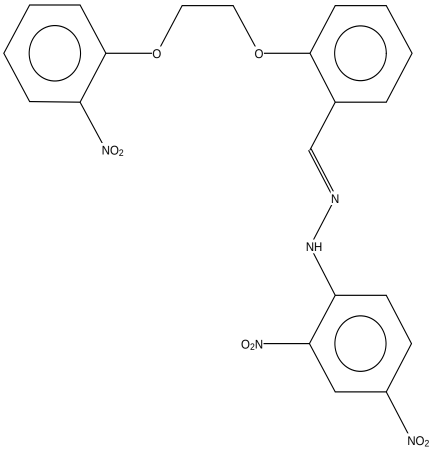

Search: search1 (Mon May 06 11:18:27 2019): Hits 17-20

ENIRAU

Reference: Shang Shan, Wei-Rong Chen, Wei-Xiao Hu, Duan-Jun Xu (2003) *Acta Crystallogr.,Sect.E:Struct.Rep.Online* ,**59,**o1723

Formula: $C_{15} H_{13} Cl_1 N_4 O_6$

Compound Name: 2-Chloro-3,4-dimethoxybenzaldehyde 2,4-dinitrophenylhydrazone

Space Group:	P21/c	***Cell:***	***a***	7.825(1)	***b***	16.145(1)	***c***	13.215(1)
Space Group No.:	14	***(Å,°)***	α	90.00	β	101.51(1)	γ	90.00
R-Factor (%):	4.50	***Temperature(K):***	293		***Density(g/cm³):***	1.546		

Parameters

Fragment 1
ANG1 (A) 6.366
DIST1 (D) 1.990

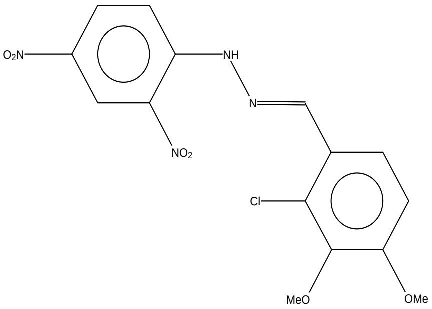

FAYDAL

Reference: Zuo-Liang Jing, Ming Yu, Xin Chen, Chun-Hua Diao, Qi-Liang Deng, Zhi Fan (2005) *Acta Crystallogr.,Sect.E:Struct.Rep.Online* ,**61,**o145

Formula: $C_{14} H_{12} N_4 O_6, C_3 H_7 N_1 O_1$

Compound Name: 2-Hydroxy-3-methoxybenzaldehyde 2,4-dinitrophenylhydrazone dimethylformamide solvate

Space Group:	P-1	***Cell:***	***a***	7.015(4)	***b***	7.759(4)	***c***	18.638(9)
Space Group No.:	2	***(Å,°)***	α	89.46(0)	β	84.37(0)	γ	68.35(0)
R-Factor (%):	5.55	***Temperature(K):***	293		***Density(g/cm³):***	1.435		

Parameters

Fragment 1
ANG1 (A) 3.302
DIST1 (D) 1.996

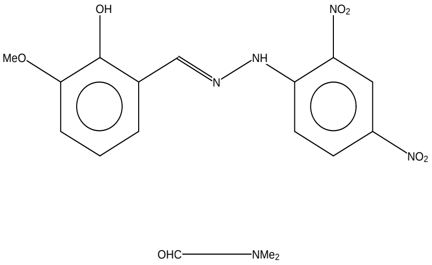

FIZFOL

Reference: H.-K.Fun, S.Chantrapromma, P.Ruanwas, T.Kobkeatthawin, C.S.Chidan Kumar (2014) *Acta Crystallogr.,Sect.E:Struct.Rep.Online* ,**70,**o89

Formula: $C_{15} H_{14} N_4 O_6$

Compound Name: (E)-1-(2,4-dinitrophenyl)-2-(3-ethoxy-4-hydroxybenzylidene)hydrazine

Space Group:	P21/c	***Cell:***	***a***	10.245(4)	***b***	13.679(5)	***c***	14.184(5)
Space Group No.:	14	***(Å,°)***	α	90.00	β	129.15(2)	γ	90.00
R-Factor (%):	4.73	***Temperature(K):***	298		***Density(g/cm³):***	1.492		

Parameters

Fragment 1
ANG1 (A) 2.247
DIST1 (D) 2.007

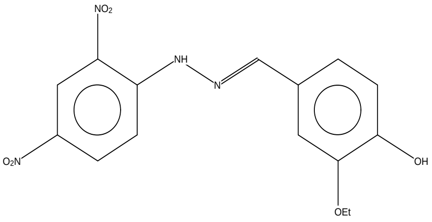

GAFTOZ

Reference: P.R.Sahoo, P.Mishra, P.Agarwal, N.Gupta, S.Kumar (2016) *J.Inclusion Phenom.Macrocyclic Chem.* ,**84,**163

Formula: $C_{20} H_{14} N_8 O_8, 2(C_2 H_6 O_1 S_1)$

Compound Name: 1,1'-(1,2-phenylenedimethylylidene)bis(2-(2,4-dinitrophenyl)hydrazine) dimethyl sulfoxide solvate

Space Group:	P21/c	***Cell:***	***a***	7.613(0)	***b***	23.924(1)	***c***	16.386(0)
Space Group No.:	14	***(Å,°)***	α	90.00	β	95.06(0)	γ	90.00
R-Factor (%):	9.42	***Temperature(K):***	293		***Density(g/cm³):***	1.454		

Parameters

Fragment 1
ANG1 (A) 12.146
DIST1 (D) 3.795

Fragment 2
ANG1 (A) 13.387
DIST1 (D) 3.841

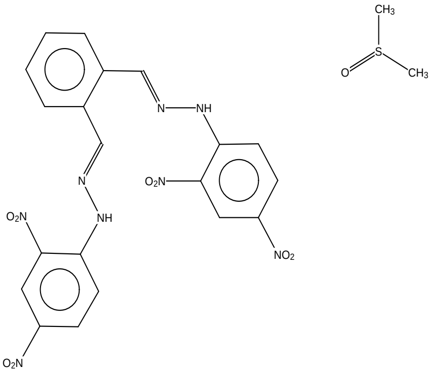

Search: search1 (Mon May 06 11:18:27 2019): Hits 21-24

GEPPOH

Reference: Jun Shi (2006) *Acta Crystallogr.,Sect.E:Struct.Rep.Online* , **62,**o4601

Formula: $C_{16} H_{16} N_4 O_6, C_3 H_7 N_1 O_1$

Compound Name: (E)-1-(3-ethoxy-4-methoxybenzylidene)-2-(2,4-dinitrophenyl)hydrazine N, N'-dimethylformamide solvate

Space Group:	P21/c	***Cell:***	***a*** 10.306(5)	***b*** 9.612(4)	***c*** 21.152(10)		
Space Group No.:	14	***(Å,°)***	α 90.00	β 90.38(0)	γ 90.00		
R-Factor (%):	4.32	***Temperature(K):***	294	***Density(g/cm3):***	1.374		

Parameters

Fragment 1
ANG1 (A) 3.469
DIST1 (D) 2.006

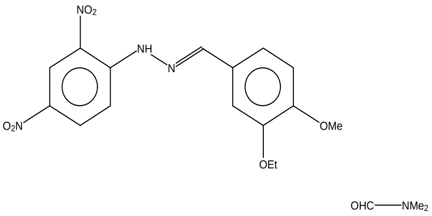

GISJAV

Reference: S.Chantrapromma, P.Ruanwas, N.Boonnak, C.S.Chidan Kumar, H.-K.Fun (2014) *Acta Crystallogr.,Sect.E:Struct.Rep.Online* ,**70,**o188

Formula: $C_{16} H_{16} N_4 O_7$

Compound Name: 1-(2,4-Dinitrophenyl)-2-[(E)-(3,4,5-trimethoxybenzylidene)hydrazine]

Space Group:	P212121	***Cell:***	***a*** 7.472(0)	***b*** 14.311(0)	***c*** 16.155(0)
Space Group No.:	19	***(Å,°)***	α 90.00	β 90.00	γ 90.00
R-Factor (%):	4.76	***Temperature(K):***	100	***Density(g/cm3):***	1.447

Parameters

Fragment 1
ANG1 (A) 5.495
DIST1 (D) 2.035

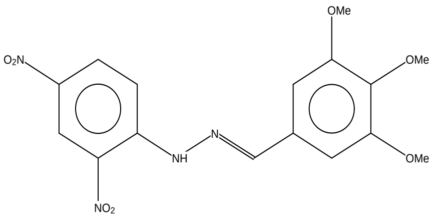

HARGOZ

Reference: Yong-Feng Cheng, Xiao-Yang Dong, Qiang-Shuai Gu, Zhang-Long Yu, Xin-Yuan Liu (2017) *Angew.Chem.,Int.Ed.* ,**56,**8883

Formula: $C_{23} H_{23} F_3 N_4 O_5$

Compound Name: 1-(2,4-dinitrophenyl)-2-({3-[3-(2,2,2-trifluoroethyl)-2-oxaspiro[4.4]nonan-3-yl]phenyl}methylidene)hydrazine

Space Group:	P1	***Cell:***	***a*** 8.490(0)	***b*** 15.629(0)	***c*** 17.579(0)
Space Group No.:	1	***(Å,°)***	α 80.24(0)	β 82.52(0)	γ 87.46(0)
R-Factor (%):	8.40	***Temperature(K):***	180	***Density(g/cm3):***	1.435

Parameters

Fragment 1
ANG1 (A) 10.142
DIST1 (D) 2.001

Fragment 2
ANG1 (A) 6.802
DIST1 (D) 2.021

Fragment 3
ANG1 (A) 8.478
DIST1 (D) 2.000

Fragment 4
ANG1 (A) 6.828
DIST1 (D) 2.001

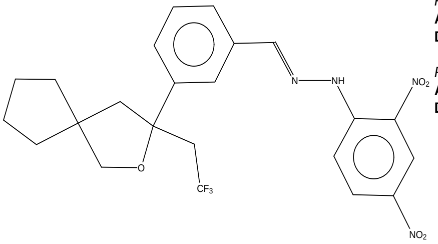

HIBCEB

Reference: D.Oehlrich, S.M.E.Vidot, M.W.Davies, G.J.Clarkson, M.Shipman (2007) *Tetrahedron* ,**63,**4703

Formula: $C_{37} H_{46} N_4 O_{10}, C_2 H_6 O_1$

Compound Name: (2S,3R,5R,6E)-5-benzyloxy-1-(3-((2,4-dinitrophenyl)hydrazonomethyl)-2,4-dihydroxy-5-isobutylphenyl)-3-hydroxy-6-(hydroxymethyl)-2-propyl-non-6-en-1-one ethanol solvate

Space Group:	P-1	***Cell:***	***a*** 12.374(1)	***b*** 13.168(1)	***c*** 13.241(1)
Space Group No.:	2	***(Å,°)***	α 83.47(0)	β 71.59(0)	γ 84.49(0)
R-Factor (%):	5.62	***Temperature(K):***	220	***Density(g/cm3):***	1.232

Parameters

Fragment 1
ANG1 (A) 10.684
DIST1 (D) 1.976

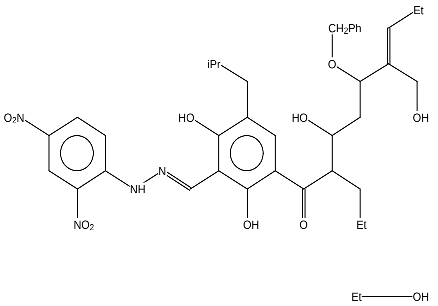

Search: search1 (Mon May 06 11:18:27 2019): Hits 25-28

HUTHOV

Reference: P.Ghosh, N.Kumar, S.K.Mukhopadhyay, P.Banerjee (2016) *Sens.Actuators,B* ,**224,**899

Formula: $C_{13} H_9 Cl_1 N_4 O_5$

Compound Name: 4-chloro-2-(((2,4-dinitrophenyl)hydrazono)methyl)phenol

Space Group:	P21/c	***Cell:***	***a*** 9.717(0)	***b*** 6.057(0)	***c*** 23.510(1)		
Space Group No.:	14	***(Å,°)***	α 90.00	β 99.04(0)	γ 90.00		
R-Factor (%):	3.06	***Temperature(K):***	293	***Density(g/cm³):***	1.636		

Parameters

Fragment 1
ANG1 (A) 3.404
DIST1 (D) 3.803

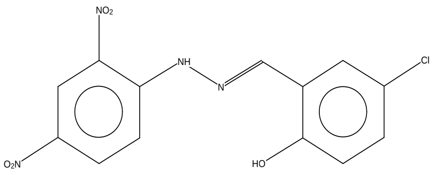

HUTHUB

Reference: P.Ghosh, N.Kumar, S.K.Mukhopadhyay, P.Banerjee (2016) *Sens.Actuators,B* ,**224,**899

Formula: $C_{14} H_{12} N_4 O_5$

Compound Name: 2-(((2,4-dinitrophenyl)hydrazono)methyl)-4-methylphenol

Space Group:	P21/n	***Cell:***	***a*** 14.016(11)	***b*** 4.489(3)	***c*** 21.970(17)		
Space Group No.:	14	***(Å,°)***	α 90.00	β 94.23(3)	γ 90.00		
R-Factor (%):	5.46	***Temperature(K):***	293	***Density(g/cm³):***	1.524		

Parameters

Fragment 1
ANG1 (A) 6.473
DIST1 (D) 1.996

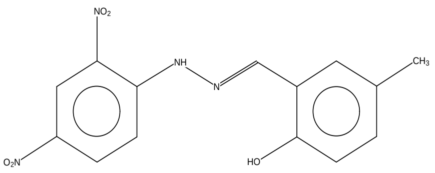

IDOGAL

Reference: J.de A.e Silva, C.Yuste-Vivas, A.J.F.N.Sobral, M.R.Silva (2013) *Acta Crystallogr.,Sect.E:Struct.Rep.Online* ,**69,**o705

Formula: $C_{21} H_{14} N_4 O_4$

Compound Name: 1-[(E)-Anthracen-9-ylmethylidene]-2-(2,4-dinitrophenyl)hydrazine

Space Group:	P212121	***Cell:***	***a*** 5.636(0)	***b*** 8.160(0)	***c*** 36.794(2)		
Space Group No.:	19	***(Å,°)***	α 90.00	β 90.00	γ 90.00		
R-Factor (%):	7.38	***Temperature(K):***	293	***Density(g/cm³):***	1.517		

Parameters

Fragment 1
ANG1 (A) 1.479
DIST1 (D) 3.829

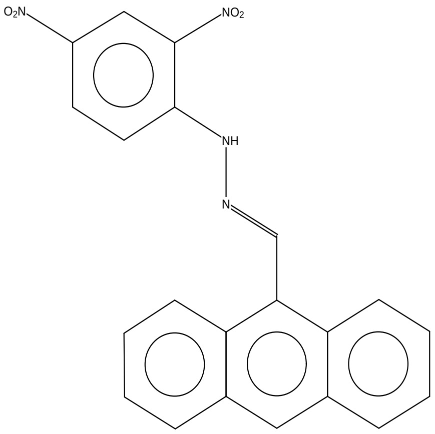

IGARUE

Reference: Zhi-Qiang Shi, Ning-Ning Ji, Xiao-Yan Li (2008) *Acta Crystallogr.,Sect.E:Struct.Rep.Online* ,**64,**o2135

Formula: $C_{13} H_9 Cl_1 N_4 O_4$

Compound Name: 1-(2-Chlorobenzylidene)-2-(2,4-dinitrophenyl)hydrazine

Space Group:	P-1	***Cell:***	***a*** 7.229(0)	***b*** 7.660(0)	***c*** 25.145(2)		
Space Group No.:	2	***(Å,°)***	α 95.69(0)	β 93.03(0)	γ 99.73(0)		
R-Factor (%):	4.51	***Temperature(K):***	293	***Density(g/cm³):***	1.564		

Parameters

Fragment 1
ANG1 (A) 3.099
DIST1 (D) 3.827

Fragment 2
ANG1 (A) 3.415
DIST1 (D) 2.000

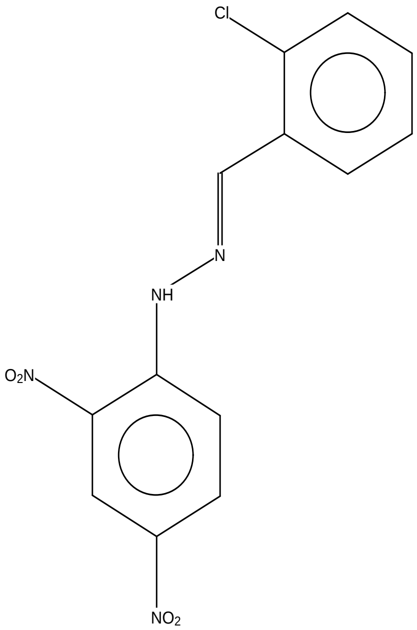

Search: search1 (Mon May 06 11:18:27 2019): Hits 29-32

INOBOC

Reference: R.G.Baughman, K.L.Martin, R.K.Singh, J.O.Stoffer (2004) *Acta Crystallogr.,Sect.C:Cryst.Struct.Commun.* ,**60,**o103

Formula: $C_{13} H_{10} N_4 O_6, C_3 H_7 N_1 O_1$

Compound Name: 2,4-Dihydroxybenzaldehyde 2,4-dinitrophenylhydrazone N,N-dimethylformamide solvate

Synonym: 4-((2,4-Dinitrophenyl)hydrazonomethyl)benzene-1,3-diol N,N-dimethylformamide solvate

Space Group:	P21/c	***Cell:***	***a*** 19.697(4)	***b*** 7.152(1)	***c*** 12.949(3)
Space Group No.:	14	***(Å,°)***	α 90.00	β 100.34(2)	γ 90.00

R-Factor (%): 5.10 ***Temperature(K):*** 293 ***Density(g/cm^3):*** 1.448

Parameters

Fragment 1
ANG1 (A) 4.106
DIST1 (D) 1.958

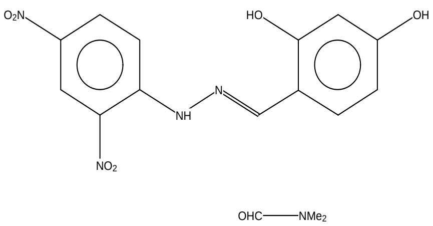

JAHPUE

Reference: A.Franken, M.J.Carr, W.Clegg, C.A.Kilner, J.D.Kennedy (2004) *Dalton Trans.* ,3552

Formula: $C_{14} H_{18} B_9 N_4 O_4{}^{1-}, C_8 H_{20} N_1{}^{1+}, C_3 H_6 O_1$

Compound Name: Tetraethylammonium 1-(4-(2,4-dinitrophenylhydrazonomethyl)phenyl)-1-carba-closo-decaborate(9) acetone solvate

Space Group:	P21/n	***Cell:***	***a*** 12.514(0)	***b*** 20.974(0)	***c*** 13.244(0)
Space Group No.:	14	***(Å,°)***	α 90.00	β 99.61(0)	γ 90.00

R-Factor (%): 8.10 ***Temperature(K):*** 173 ***Density(g/cm^3):*** 1.147

Parameters

Fragment 1
ANG1 (A) 11.559
DIST1 (D) 1.872

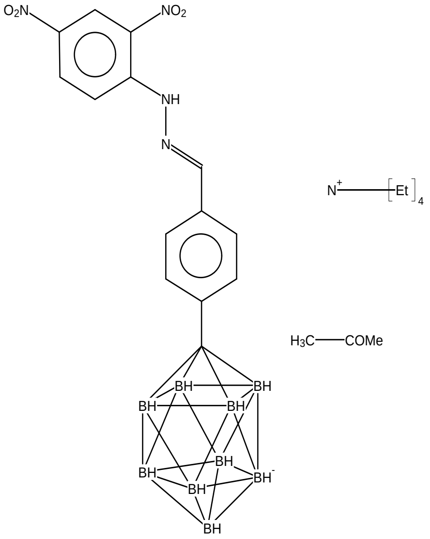

KABCOH

Reference: Lin-xiu Zhao, Gang-shen Li (2010) *Acta Crystallogr.,Sect.E:Struct.Rep.Online* ,**66,**o2644

Formula: $C_{15} H_{14} N_4 O_7, C_3 H_7 N_1 O_1, 0.5(H_2 O_1)$

Compound Name: 4-hydroxy-3,5-dimethoxybenzaldehyde (2,4-dinitrophenyl)hydrazone N,N-dimethylformamide solvate hemihydrate

Synonym: 4-(2,4-dinitrobenzenecarbohydrazonoyl)-2,6-dimethoxyphenol N,N-dimethylformamide solvate hemihydrate

Space Group:	P-1	***Cell:***	***a*** 12.208(2)	***b*** 13.931(2)	***c*** 14.537(2)
Space Group No.:	2	***(Å,°)***	α 62.41(1)	β 74.41(1)	γ 72.17(1)

R-Factor (%): 3.49 ***Temperature(K):*** 293 ***Density(g/cm^3):*** 1.431

Parameters

Fragment 1
ANG1 (A) 6.056
DIST1 (D) 2.047

Fragment 2
ANG1 (A) 7.744
DIST1 (D) 2.056

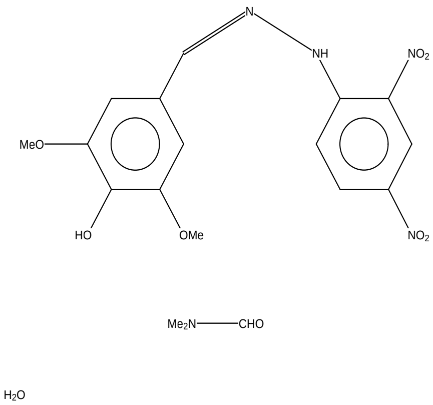

KEQRAA

Reference: Xin Chen, Ming Yu (2006) *Acta Crystallogr.,Sect.E:Struct.Rep.Online* ,**62,**o4466

Formula: $C_{21} H_{18} N_4 O_8 S_1$

Compound Name: (E)-1-(2,4-Dinitrophenyl)-2-(3-methoxy-4-(p-tolylsulfonyloxy)benzylidene) hydrazine

Space Group:	P-1	***Cell:***	***a*** 7.642(1)	***b*** 9.533(2)	***c*** 15.727(4)
Space Group No.:	2	***(Å,°)***	α 105.32(0)	β 96.56(0)	γ 95.35(0)

R-Factor (%): 4.81 ***Temperature(K):*** 294 ***Density(g/cm^3):*** 1.484

Parameters

Fragment 1
ANG1 (A) 5.072
DIST1 (D) 2.002

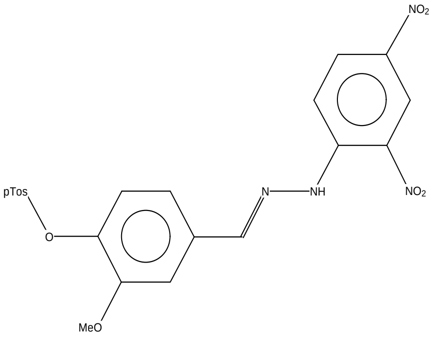

Search: search1 (Mon May 06 11:18:27 2019): Hits 33-36

KERSIK

Reference: Chun-Hua Diao, Xin Chen (2006) *Acta Crystallogr.,Sect.E:Struct.Rep.Online* ,**62,**o4896

Formula: $C_{22}\ H_{18}\ N_6\ O_{11}$

Compound Name: (E)-2-(2-(2,4-dinitrophenoxy)ethoxy)-3-methoxybenzaldehyde 2,4-dinitrophenylhydrazone

Space Group:	P-1	***Cell:***	***a***	9.447(2)	***b***	10.286(2)	***c***	13.357(3)
Space Group No.:	2	***(Å,°)***	α	68.91(0)	β	76.70(0)	γ	85.64(0)

R-Factor (%): 4.44 ***Temperature(K):*** 294 ***Density(g/cm^3):*** 1.529

Parameters

Fragment 1
ANG1 (A) 2.638
DIST1 (D) 3.818

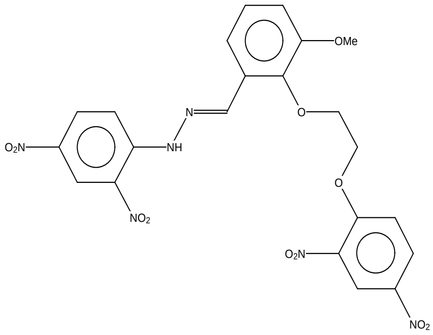

KERTUX

Reference: Jun Shi (2006) *Acta Crystallogr.,Sect.E:Struct.Rep.Online* , **62,**o5337

Formula: $C_{17}\ H_{17}\ Br_1\ N_4\ O_6,C_3\ H_7\ N_1\ O_1$

Compound Name: (E)-1-(5-Bromo-3-methoxy-2-propoxybenzylidene)-2-(2,4-dinitrophenyl) hydrazine N,N-dimethylformamide solvate

Space Group:	P-1	***Cell:***	***a***	8.044(4)	***b***	11.803(6)	***c***	12.735(6)
Space Group No.:	2	***(Å,°)***	α	106.82(0)	β	94.16(0)	γ	94.75(0)

R-Factor (%): 4.15 ***Temperature(K):*** 294 ***Density(g/cm^3):*** 1.523

Parameters

Fragment 1
ANG1 (A) 4.285
DIST1 (D) 2.028

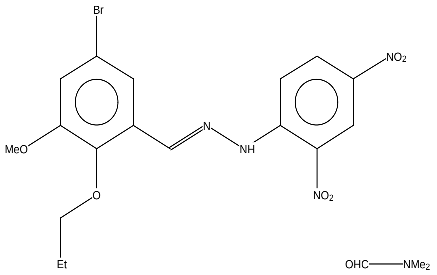

KOFGAO

Reference: Feng-yu Bao (2008) *Acta Crystallogr.,Sect.E:Struct.Rep.Online* ,**64,**o1433

Formula: $C_{13}\ H_8\ Cl_2\ N_4\ O_4$

Compound Name: 2,4-Dichlorobenzaldehyde 2,4-dinitrophenylhydrazone

Space Group:	P21/c	***Cell:***	***a***	13.381(0)	***b***	28.998(1)	***c***	7.400(0)
Space Group No.:	14	***(Å,°)***	α	90.00	β	92.42(0)	γ	90.00

R-Factor (%): 6.12 ***Temperature(K):*** 291 ***Density(g/cm^3):*** 1.645

Parameters

Fragment 1
ANG1 (A) 3.721
DIST1 (D) 3.866

Fragment 2
ANG1 (A) 4.395
DIST1 (D) 2.045

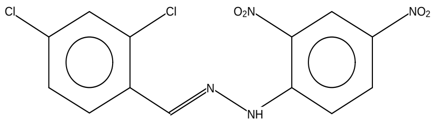

LENDAM

Reference: E.Sahin, A.menzek (2017) *CSD Communication(Private Communication)* ,

Formula: $C_{20}\ H_{16}\ N_4\ O_4$

Compound Name: 1-(2,4-dinitrophenyl)-2-{[2-(prop-2-en-1-yl)naphthalen-1-yl]methylidene}hydrazine

Space Group:	C2/c	***Cell:***	***a***	12.101(1)	***b***	8.405(0)	***c***	34.768(3)
Space Group No.:	15	***(Å,°)***	α	90.00	β	95.52(0)	γ	90.00

R-Factor (%): 5.64 ***Temperature(K):*** 293 ***Density(g/cm^3):*** 1.420

Parameters

Fragment 1
ANG1 (A) 3.478
DIST1 (D) 2.008

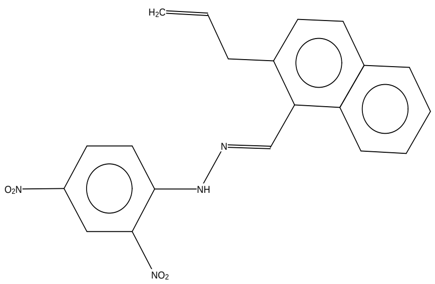

Search: search1 (Mon May 06 11:18:27 2019): Hits 37-40

LEPSUV

Reference: Xin Chen, Ming Yu (2006) *Acta Crystallogr.,Sect.E:Struct.Rep.Online* ,**62,**o4468

Formula: $C_{21} H_{18} N_4 O_8 S_1$

Compound Name: (E)-1-(2,4-Dinitrophenyl)-2-(3-methoxy-2-(p-tolylsulfonyloxy)benzylidene) hydrazine

Space Group:	P21/c	***Cell:***	***a*** 7.983(1)	***b*** 16.545(3)	***c*** 16.664(3)		
Space Group No.:	14	***(Å,°)***	α 90.00	β 101.56(0)	γ 90.00		
R-Factor (%):	4.27	***Temperature(K):***	294	***Density(g/cm^3):***	1.498		

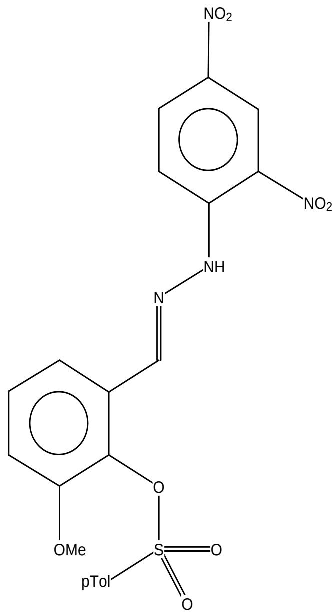

Parameters

Fragment 1
ANG1 (A) 7.521
DIST1 (D) 2.039

LUKLUY

Reference: M.P.Groziak, P.D.Robinson (2002) *Collect.Czech.Chem.Commun.* ,**67,**1084

Formula: $C_{18} H_{21} B_1 N_4 O_7$

Compound Name: Diethyl 2-((2,4-dinitrophenyl)hydrazonomethyl)-4-methoxybenzeneboronate

Space Group:	P21/n	***Cell:***	***a*** 9.443(2)	***b*** 15.436(2)	***c*** 13.982(3)		
Space Group No.:	14	***(Å,°)***	α 90.00	β 104.80(1)	γ 90.00		
R-Factor (%):	5.20	***Temperature(K):***	296	***Density(g/cm^3):***	1.403		

Parameters

Fragment 1
ANG1 (A) 20.716
DIST1 (D) 2.023

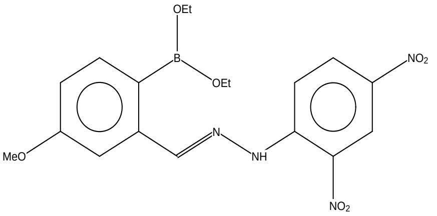

LUKMAF

Reference: M.P.Groziak, P.D.Robinson (2002) *Collect.Czech.Chem.Commun.* ,**67,**1084

Formula: $C_{17} H_{19} B_1 N_4 O_6$

Compound Name: Diethyl (2-((2,4-dinitrophenylhydrazono)methyl)phenyl)boronate

Space Group:	P-1	***Cell:***	***a*** 11.194(3)	***b*** 12.225(4)	***c*** 8.185(1)		
Space Group No.:	2	***(Å,°)***	α 93.32(2)	β 107.33(1)	γ 64.68(3)		
R-Factor (%):	4.90	***Temperature(K):***	296	***Density(g/cm^3):***	1.331		

Parameters

Fragment 1
ANG1 (A) 1.494
DIST1 (D) 2.003

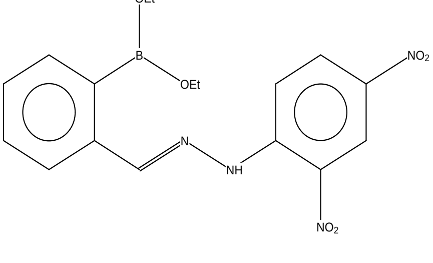

MEBCUS

Reference: Chun-Hua Diao, Zhi Fan, Ming Yu, Xin Chen, Zuo-Liang Jing (2006) *Acta Crystallogr.,Sect.E:Struct.Rep.Online* ,**62,** o39

Formula: $C_{21} H_{17} N_5 O_8,C_3 H_7 N_1 O_1$

Compound Name: (E)-1-(2,4-Dinitrophenyl)-2-(2-(2-(3-nitrophenoxy)ethoxy)benzylidene) hydrazine N,N-dimethylformamide solvate

Space Group:	P21/c	***Cell:***	***a*** 6.981(1)	***b*** 25.764(4)	***c*** 14.200(2)		
Space Group No.:	14	***(Å,°)***	α 90.00	β 96.32(0)	γ 90.00		
R-Factor (%):	5.23	***Temperature(K):***	294	***Density(g/cm^3):***	1.414		

Parameters

Fragment 1
ANG1 (A) 4.722
DIST1 (D) 2.011

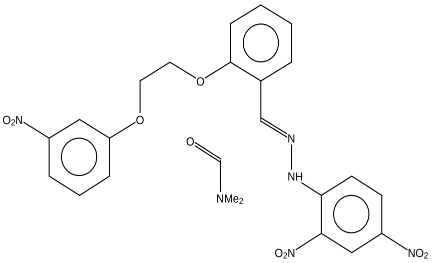

Search: search1 (Mon May 06 11:18:27 2019): Hits 41-44

MEGVIE

Reference: Zuo-Liang Jing, Jia-Cui Sun, Xin Chen, Yu Ming (2006)
Acta Crystallogr.,Sect.E:Struct.Rep.Online ,**62,**o1202

Formula: $C_{13} H_{10} N_4 O_5,C_3 H_7 N_1 O_1$

Compound Name: 3-((2,4-Dinitrophenyl)hydrazonomethyl)phenol dimethylformamide solvate

Space Group:	P2/c	***Cell:***	***a***	17.701(4)	***b***	7.066(1)	***c***	14.695(4)
Space Group No.:	13	***(Å,°)***	α	90.00	β	104.61(0)	γ	90.00

R-Factor (%): 3.99 ***Temperature(K):*** 294 ***Density(g/cm³):*** 1.402

Parameters

Fragment 1
ANG1 (A) 2.456
DIST1 (D) 2.008

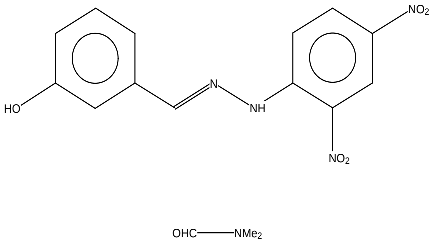

NEQKUQ

Reference: Qiao-Zhen Zhang, Yan-Li Zhao, Xin Chen, Ming Yu (2006)
Acta Crystallogr.,Sect.E:Struct.Rep.Online ,**62,**o4668

Formula: $C_{21} H_{18} N_4 O_8 S_1$

Compound Name: (E)-1-(2,4-Dinitrophenyl)-2-(4-methoxy-3-(4-methylbenzenesulfonyloxy) benzylidene)hydrazine

Space Group:	P-1	***Cell:***	***a***	7.365(2)	***b***	12.978(4)	***c***	13.547(4)
Space Group No.:	2	***(Å,°)***	α	62.94(0)	β	88.90(0)	γ	74.84(0)

R-Factor (%): 4.87 ***Temperature(K):*** 294 ***Density(g/cm³):*** 1.461

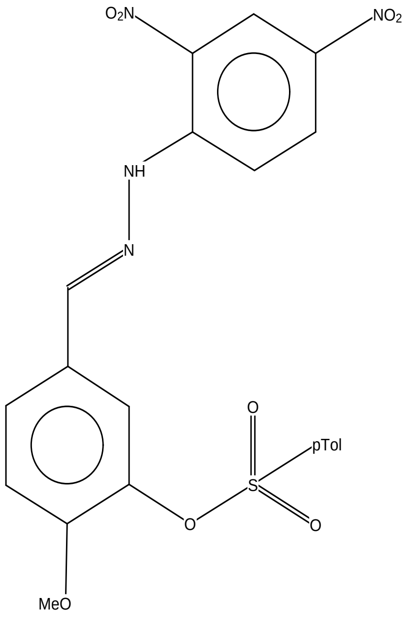

Parameters

Fragment 1
ANG1 (A) 3.735
DIST1 (D) 2.040

NEQLAX

Reference: Yan-Li Zhao, Qiao-Zhen Zhang, Xin Chen, Ming Yu (2006)
Acta Crystallogr.,Sect.E:Struct.Rep.Online ,**62,**o4734

Formula: $C_{20} H_{15} Cl_1 N_4 O_5,C_3 H_7 N_1 O_1$

Compound Name: (Z)-1-(2-(2-Chlorobenzyloxy)benzylidene)-2-(2,4-dinitrophenyl)hydrazine N,N-dimethylformamide solvate

Space Group:	P21/c	***Cell:***	***a***	7.844(4)	***b***	12.887(7)	***c***	23.631(13)
Space Group No.:	14	***(Å,°)***	α	90.00	β	92.45(0)	γ	90.00

R-Factor (%): 4.38 ***Temperature(K):*** 294 ***Density(g/cm³):*** 1.391

Parameters

Fragment 1
ANG1 (A) 4.994
DIST1 (D) 2.052

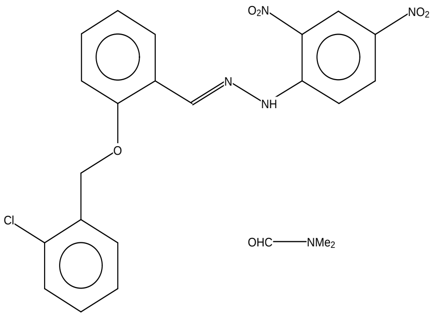

NERVAI

Reference: Jun Shi (2006) *Acta Crystallogr.,Sect.E:Struct.Rep.Online* ,
62,o5848

Formula: $C_{21} H_{18} N_4 O_6,C_3 H_7 N_1 O_1$

Compound Name: (E)-1-(2-(Benzyloxy)-3-methoxybenzylidene)-2-(2,4-dinitrophenyl) hydrazine N,N-dimethylformamide solvate

Space Group:	P-1	***Cell:***	***a***	8.244(3)	***b***	11.792(4)	***c***	12.789(4)
Space Group No.:	2	***(Å,°)***	α	86.26(0)	β	87.31(0)	γ	86.86(0)

R-Factor (%): 5.12 ***Temperature(K):*** 294 ***Density(g/cm³):*** 1.330

Parameters

Fragment 1
ANG1 (A) 5.256
DIST1 (D) 3.898

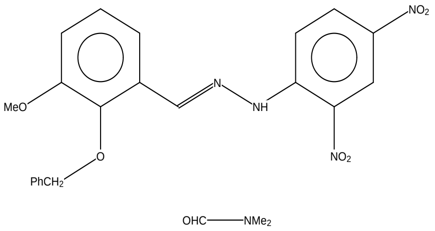

Search: search1 (Mon May 06 11:18:27 2019): Hits 45-48

NOBFAM

Reference: N.Chitrapriya, V.Mahalingam, M.Zeller, K.Natarajan (2008) *Polyhedron* ,**27,**1573

Formula: $C_{50}\,H_{38}\,N_4\,O_6\,P_2\,Ru_1,C_3\,H_7\,N_1\,O_1$

Compound Name: Carbonyl-(2-(2-(2-oxybenzylidene)hydrazinyl)-3,5-dinitrophenyl-C,N,O)-bis(triphenylphosphine)-ruthenium(ii) N,N-dimethylformamide solvate

Space Group:	P21/n	***Cell:***	***a*** 11.750(1)	***b*** 26.274(2)	***c*** 16.139(1)
Space Group No.:	14	***(Å,°)***	α 90.00	β 98.85(0)	γ 90.00

R-Factor (%): 4.82 ***Temperature(K):*** 100 ***Density(g/cm^3):*** 1.386

Parameters

Fragment 1
ANG1 (A) 17.101
DIST1 (D) 3.873

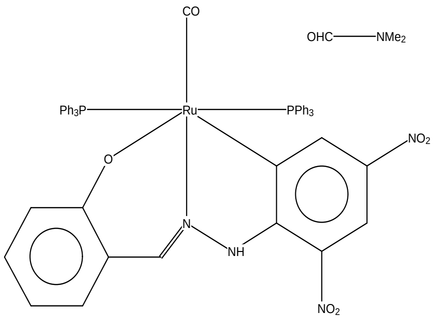

OBOHOF

Reference: M.S.H.Faizi, N.Dege, A.Haque, V.A.Kalibabchuk, M.Cemberci (2017) *Acta Crystallogr.,Sect.E:Cryst.Commun.* ,**73,**96

Formula: $C_{21}\,H_{26}\,N_4\,O_5$

Compound Name: 2,6-di-t-butyl-4-(((2,4-dinitrophenyl)hydrazono)methyl)phenol

Space Group:	Pnma	***Cell:***	***a*** 18.765(1)	***b*** 6.919(0)	***c*** 17.259(1)
Space Group No.:	62	***(Å,°)***	α 90.00	β 90.00	γ 90.00

R-Factor (%): 7.12 ***Temperature(K):*** 296 ***Density(g/cm^3):*** 1.228

Parameters

Fragment 1
ANG1 (A) 0.000
DIST1 (D) 1.956

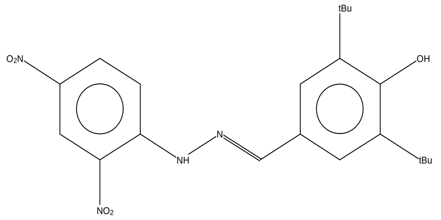

OKOMAE

Reference: Xun Feng, Ai-Qin Tian, Cai-Lu Wang, Yang Wang (2016) *Z.Kristallogr.-New Cryst.Struct.* ,**231,**37

Formula: $2(C_{13}\,H_9\,N_5\,O_7),C_6\,H_{15}\,N_1$

Compound Name: bis(2-(((2,4-dinitrophenyl)hydrazono)methyl)-4-nitrophenol) triethylamine

Space Group:	P21/c	***Cell:***	***a*** 12.334(1)	***b*** 37.764(5)	***c*** 8.208(1)
Space Group No.:	14	***(Å,°)***	α 90.00	β 109.05(0)	γ 90.00

R-Factor (%): 6.22 ***Temperature(K):*** 296 ***Density(g/cm^3):*** 1.463

Parameters

Fragment 1
ANG1 (A) 13.616
DIST1 (D) 3.807

Fragment 2
ANG1 (A) 1.542
DIST1 (D) 3.915

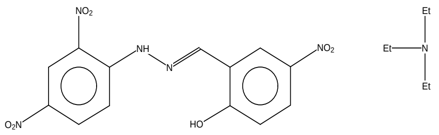

OMOLAD

Reference: C.Glidewell, J.N.Low, J.M.S.Skakle, J.L.Wardell (2004) *Acta Crystallogr.,Sect.C:Cryst.Struct.Commun.* ,**60,**o19

Formula: $C_{13}\,H_9\,I_1\,N_4\,O_4$

Compound Name: 3-Iodobenzaldehyde 2,4-dinitrophenylhydrazone

Space Group:	C2/c	***Cell:***	***a*** 30.060(0)	***b*** 13.563(0)	***c*** 7.026(0)
Space Group No.:	15	***(Å,°)***	α 90.00	β 101.39(0)	γ 90.00

R-Factor (%): 2.99 ***Temperature(K):*** 120 ***Density(g/cm^3):*** 1.950

Parameters

Fragment 1
ANG1 (A) 7.570
DIST1 (D) 1.980

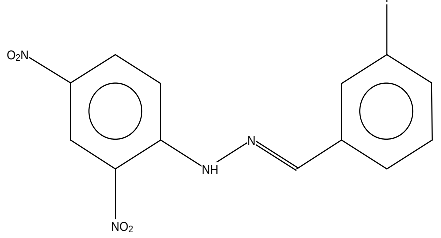

OMOLEH

Reference: C.Glidewell, J.N.Low, J.M.S.Skakle, J.L.Wardell (2004)
Acta Crystallogr.,Sect.C:Cryst.Struct.Commun. ,**60,**o19

Formula: $C_{13}\ H_9\ I_1\ N_4\ O_4$

Compound Name: 4-Iodobenzaldehyde 2,4-dinitrophenylhydrazone

Space Group:	C2/c	***Cell:***	***a*** 32.359(2)	***b*** 4.629(0)	***c*** 18.417(1)
Space Group No.:	15	***(Å,°)***	α 90.00	β 97.63(0)	γ 90.00
R-Factor (%):	4.64	***Temperature(K):***	120	***Density(g/cm^3):***	2.002

Parameters

Fragment 1
ANG1 (A) 9.332
DIST1 (D) 2.036

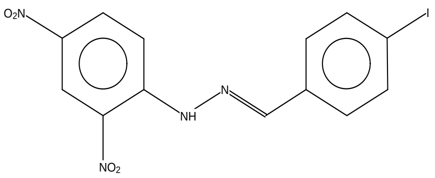

PONTIW

Reference: H.-K.Fun, R.Kia, H.Kargar (2009)
Acta Crystallogr.,Sect.E:Struct.Rep.Online ,**65,**o246

Formula: $C_{14}\ H_{12}\ N_4\ O_5$

Compound Name: 2-Methoxybenzaldehyde 2,4-dinitrophenylhydrazone

Space Group:	P-1	***Cell:***	***a*** 7.032(0)	***b*** 7.621(0)	***c*** 14.190(0)
Space Group No.:	2	***(Å,°)***	α 98.05(0)	β 97.06(0)	γ 109.47(0)
R-Factor (%):	4.46	***Temperature(K):***	100	***Density(g/cm^3):***	1.505

Parameters

Fragment 1
ANG1 (A) 3.908
DIST1 (D) 2.029

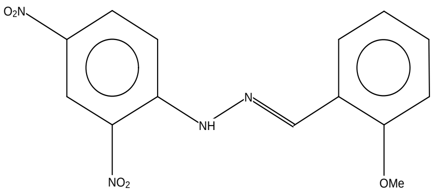

PUBWOZ

Reference: You-Ming Zhang, Qi Lin, Tai-Bao Wei, Dan-Dan Wang,
Hong Yao, Ya-Lin Wang (2009) *Sens.Actuators,B* ,**137,**447

Formula: $C_{20}\ H_{13}\ Cl_1\ N_8\ O_9,2(C_2\ H_6\ O_1\ S_1),0.5(H_2\ O_1)$

Compound Name: 4-Chloro-2,6-bis((2-(2,4-dinitrophenyl)hydrazono)methyl)phenol dimethylsulfoxide solvate hemihydrate

Space Group:	C2/c	***Cell:***	***a*** 30.524(6)	***b*** 13.952(2)	***c*** 30.386(5)
Space Group No.:	15	***(Å,°)***	α 90.00	β 94.49(0)	γ 90.00
R-Factor (%):	9.56	***Temperature(K):***	294	***Density(g/cm^3):***	1.462

Parameters

Fragment 1
ANG1 (A) 6.466
DIST1 (D) 3.830

Fragment 2
ANG1 (A) 8.458
DIST1 (D) 2.009

Fragment 3
ANG1 (A) 0.677
DIST1 (D) 2.034

Fragment 4
ANG1 (A) 3.329
DIST1 (D) 2.011

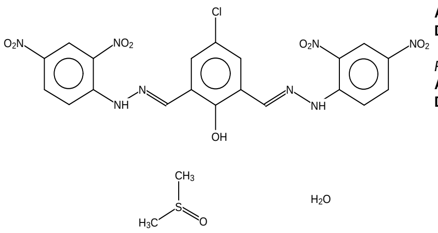

PUZSUZ

Reference: O.Kundrat, H.Dvorakova, V.Eigner, P.Lhotak (2010)
J.Org.Chem. ,**75,**407

Formula: $C_{43}\ H_{44}\ N_4\ O_8\ S_4$

Compound Name: 1-(2,4-Dinitrophenyl)-2-((25,26,27,28-tetrapropoxy-2,8,14,20-tetrathiapentacyclo[19.3.1.1^{3,7}.1^{9,13}.1^{15,19}]octacosa-1(25),3(28),4,6,9(27),10,12,15(26),16,18,21,23-dodecaen-4-yl)methylene)hydrazine

Synonym: 4-(2,4-Dinitrophenylhydrazinylidenemethyl)-25,26,27,28-tetra-n-propoxy-2,8,14,20-tetrathiacalix(4)arene

Space Group:	C2/c	***Cell:***	***a*** 32.859(3)	***b*** 16.839(1)	***c*** 15.970(1)
Space Group No.:	15	***(Å,°)***	α 90.00	β 102.53(1)	γ 90.00
R-Factor (%):	6.90	***Temperature(K):***	150	***Density(g/cm^3):***	1.345

Parameters

Fragment 1
ANG1 (A) 21.425
DIST1 (D) 2.012

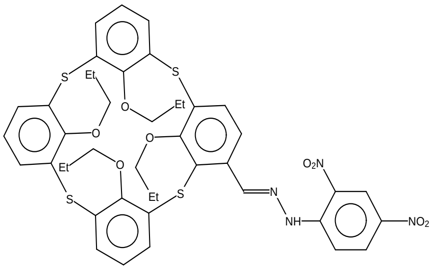

Search: search1 (Mon May 06 11:18:27 2019): Hits 53-56

QAVXER

Reference: S.J.O'Malley, K.L.Tan, A.Watzke, R.G.Bergman, J.A.Ellman (2005) *J.Am.Chem.Soc.* ,**127,**13496

Formula: $C_{26}\ H_{23}\ Br_1\ N_4\ O_{10}$,$C_1\ H_2\ Cl_2$

Compound Name: Methyl 2-(2-bromo-4,5-dimethoxyphenyl)-4-((2,4-dinitrophenyl) hydrazonomethyl)-7-methoxy-2,3-dihydrobenzofuran-3-carboxylate dichloromethane solvate

Space Group:	P212121	***Cell:***	***a***	6.543(0)	***b***	14.702(0)	***c***	31.477(2)
Space Group No.:	19	***(Å,°)***	α	90.00	β	90.00	γ	90.00

R-Factor (%): 3.79 ***Temperature(K):*** 143 ***Density(g/cm3):*** 1.571

Parameters

Fragment 1
ANG1 (A) 9.345
DIST1 (D) 2.011

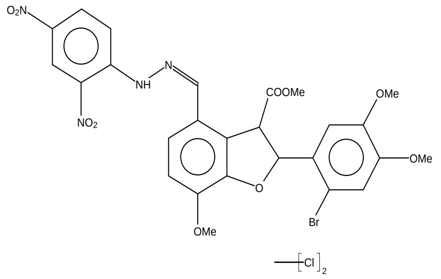

RUFSOB

Reference: G.V.Gurskaya, V.E.Zavodnik, N.E.Zhukhlistova, M.V.Kozlov (2008) *Kristallografiya(Russ.)(Crystallogr.Rep.)* ,**53,**663

Formula: $C_{13}\ H_{10}\ N_4\ O_6$

Compound Name: 2,4-Dinitrophenylhydrazone 2,3-dihydroxybenzaldehyde

Synonym: 3-((2,4-Dinitrophenyl)carbonohydrazonoyl)benzene-1,2-diol

Space Group:	P21/n	***Cell:***	***a***	9.158(1)	***b***	6.289(1)	***c***	22.844(3)
Space Group No.:	14	***(Å,°)***	α	90.00	β	94.67(0)	γ	90.00

R-Factor (%): 4.71 ***Temperature(K):*** 120 ***Density(g/cm3):*** 1.612

Parameters

Fragment 1
ANG1 (A) 2.232
DIST1 (D) 3.775

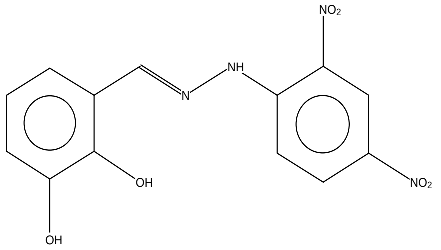

SUYPEI

Reference: Lin-xiu Zhao, Duan-lin Cao, Jian-lan Cui (2010) *Acta Crystallogr.,Sect.E:Struct.Rep.Online* ,**66,**o2204

Formula: $C_{14}\ H_{12}\ N_4\ O_6$,$C_5\ H_5\ N_1$

Compound Name: 2-Hydroxy-3-methoxybenzaldehyde 2,4-dinitrophenylhydrazone pyridine solvate

Space Group:	P-1	***Cell:***	***a***	6.902(1)	***b***	7.624(1)	***c***	19.073(3)
Space Group No.:	2	***(Å,°)***	α	95.11(1)	β	91.20(1)	γ	107.02(1)

R-Factor (%): 5.13 ***Temperature(K):*** 293 ***Density(g/cm3):*** 1.431

Parameters

Fragment 1
ANG1 (A) 3.486
DIST1 (D) 2.033

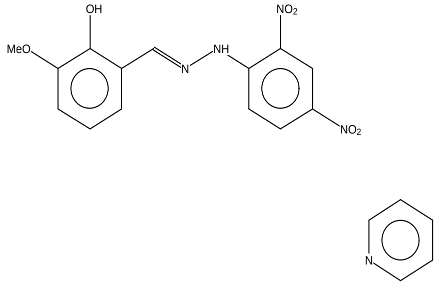

SUYPIM

Reference: Lin-xiu Zhao, Jian-lan Cui, Duan-lin Cao (2010) *Acta Crystallogr.,Sect.E:Struct.Rep.Online* ,**66,**o2205

Formula: $C_{15}\ H_{14}\ N_4\ O_6$,$C_3\ H_7\ N_1\ O_1$

Compound Name: 3-Ethoxy-2-hydroxybenzaldehyde 2,4-dinitrophenylhydrazone N,N-dimethylformamide solvate

Space Group:	P-1	***Cell:***	***a***	7.107(0)	***b***	7.720(0)	***c***	19.479(1)
Space Group No.:	2	***(Å,°)***	α	84.68(0)	β	81.56(0)	γ	68.71(0)

R-Factor (%): 3.94 ***Temperature(K):*** 293 ***Density(g/cm3):*** 1.415

Parameters

Fragment 1
ANG1 (A) 3.049
DIST1 (D) 2.015

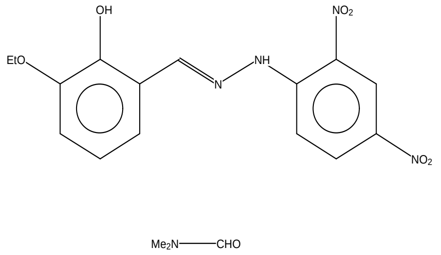

Search: search1 (Mon May 06 11:18:27 2019): Hits 57-60

UREDOL

Reference: Xianrong Xin, Min Li, Zhimin Chen, Ruitao Zhu (2011) *Acta Crystallogr.,Sect.E:Struct.Rep.Online* ,**67,**o1169

Formula: C_{15} H_{14} N_4 O_6

Compound Name: 1-(2,3-Dimethoxybenzylidene)-2-(2,4-dinitrophenyl)hydrazine

Space Group: P-1 **Cell:** **a** 7.841(0) **b** 7.920(0) **c** 13.896(1)
Space Group No.: 2 **(Å,°)** α 85.04(0) β 82.77(0) γ 65.89(0)

R-Factor (%): 6.33 **Temperature(K):** 298 **Density(g/cm³):** 1.473

Parameters

Fragment 1
ANG1 (A) 3.690
DIST1 (D) 1.989

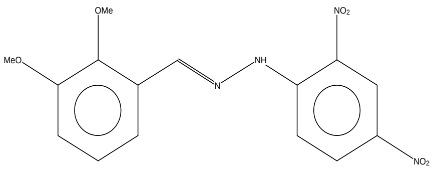

UREKIM

Reference: J.P.Jasinski, A.N.Braley, C.S.C.Kumar, H.S.Yathirajan, A.N.Mayekar (2011) *Acta Crystallogr.,Sect.E:Struct.Rep.Online* ,**67,** o1200

Formula: C_{13} H_9 F_1 N_4 O_4

Compound Name: (E)-1-(2,4-Dinitrophenyl)-2-(2-fluorobenzylidene)hydrazine

Space Group: P-1 **Cell:** **a** 7.096(0) **b** 8.271(0) **c** 11.723(0)
Space Group No.: 2 **(Å,°)** α 88.61(0) β 80.54(0) γ 71.37(1)

R-Factor (%): 4.54 **Temperature(K):** 173 **Density(g/cm³):** 1.572

Parameters

Fragment 1
ANG1 (A) 5.670
DIST1 (D) 2.019

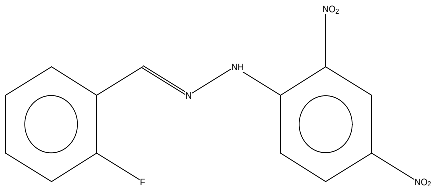

WACROJ

Reference: Lin-xiu Zhao, Gang-shen Li (2010) *Acta Crystallogr.,Sect.E:Struct.Rep.Online* ,**66,**o3108

Formula: C_{17} H_{19} N_5 O_5

Compound Name: 5-Diethylamino-2-((2-(2,4-dinitrophenyl)hydrazin-1-ylidene)methyl) phenol

Space Group: P-1 **Cell:** **a** 8.530(0) **b** 8.541(0) **c** 12.491(1)
Space Group No.: 2 **(Å,°)** α 84.55(0) β 89.73(0) γ 75.11(0)

R-Factor (%): 5.51 **Temperature(K):** 293 **Density(g/cm³):** 1.417

Parameters

Fragment 1
ANG1 (A) 1.773
DIST1 (D) 3.845

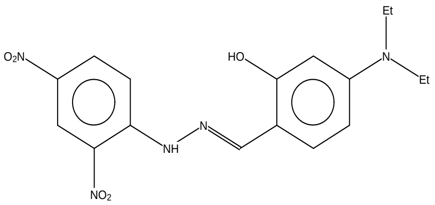

WAFSAZ

Reference: Xiu-Rong Zhai (2010) *Acta Crystallogr.,Sect.E:Struct.Rep.Online* ,**66,**o3142

Formula: C_{13} H_{10} N_4 O_5,0.5(C_2 H_6 O_1)

Compound Name: 4-([2-(2,4-Dinitrophenyl)hydrazinylidene]methyl)phenol ethanol solvate

Synonym: 4-(2,4-Dinitrobenzenecarbohydrazonoyl)phenol ethanol solvate

Space Group: P-1 **Cell:** **a** 7.094(1) **b** 7.289(1) **c** 14.458(3)
Space Group No.: 2 **(Å,°)** α 100.16(0) β 96.38(0) γ 100.60(0)

R-Factor (%): 6.41 **Temperature(K):** 295 **Density(g/cm³):** 1.510

Parameters

Fragment 1
ANG1 (A) 4.292
DIST1 (D) 3.818

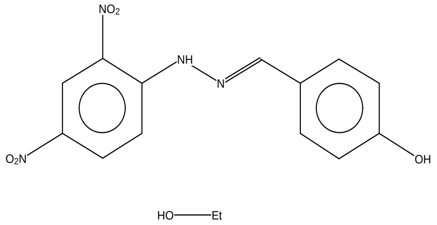

Search: search1 (Mon May 06 11:18:27 2019): Hits 61-64

WALLEA

Reference: J.Granell, R.Moragas, J.Sales, M.Font-Bardia, X.Solans (1993) *J.Chem.Soc.,Dalton Trans.* ,1237

Formula: $C_{34} H_{30} Br_1 N_4 O_4 P_1 Pd_1, C_1 H_2 Cl_2$

Compound Name: (2,4,6-Trimethylbenzylidene(4,6-dinitro-o-phenylene)hydrazone-C,N)-triphenylphosphine-bromo-palladium dichloromethane solvate

Space Group:	P-1	***Cell:***	***a*** 9.429(2)	***b*** 10.420(2)	***c*** 18.334(3)		
Space Group No.:	2	***(Å,°)***	α 92.23(3)	β 90.26(3)	γ 96.11(2)		
R-Factor (%):	6.20	***Temperature(K):***	295	***Density(g/cm³):***	1.597		

Parameters

Fragment 1
ANG1 (A) 74.159
DIST1 (D) 1.813

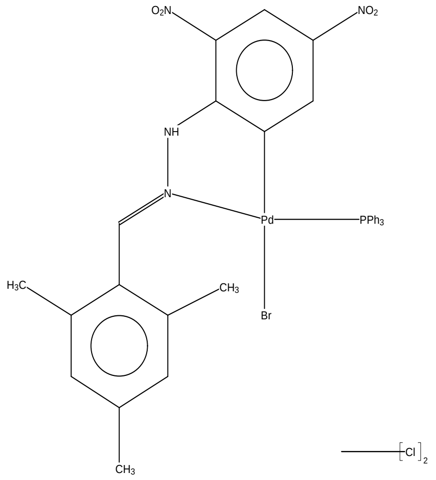

WUXCUN

Reference: C.Glidewell, J.N.Low, J.M.S.Skakle, J.L.Wardell (2003) *Acta Crystallogr.,Sect.C:Cryst.Struct.Commun.* ,**59,**o98

Formula: $C_{13} H_9 I_1 N_4 O_4$

Compound Name: 1-(2-Iodobenzylidene)-2-(2,4-dinitrophenyl)hydrazone

Space Group:	P-1	***Cell:***	***a*** 7.889(0)	***b*** 8.141(0)	***c*** 12.176(1)		
Space Group No.:	2	***(Å,°)***	α 83.88(0)	β 83.68(0)	γ 63.15(0)		
R-Factor (%):	4.70	***Temperature(K):***	120	***Density(g/cm³):***	1.978		

Parameters

Fragment 1
ANG1 (A) 9.907
DIST1 (D) 2.063

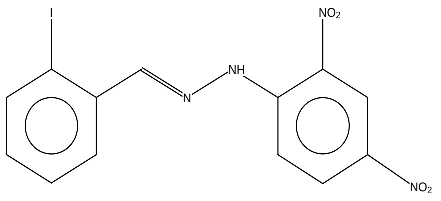

WUXHAY

Reference: Shang Shan, Duan-Jun Xu, Chen-Hsiung Hung, Jing-Yun Wu, M.Y.Chiang (2003) *Acta Crystallogr.,Sect.C:Cryst.Struct.Commun.* ,**59,**o135

Formula: $C_{13} H_{10} N_4 O_4$

Compound Name: 1-Benzylidene-2-(2,4-dinitrophenyl)hydrazone

Space Group:	P21/c	***Cell:***	***a*** 13.290(3)	***b*** 6.825(3)	***c*** 14.332(1)		
Space Group No.:	14	***(Å,°)***	α 90.00	β 92.60(1)	γ 90.00		
R-Factor (%):	4.00	***Temperature(K):***	298	***Density(g/cm³):***	1.464		

Parameters

Fragment 1
ANG1 (A) 3.262
DIST1 (D) 3.860

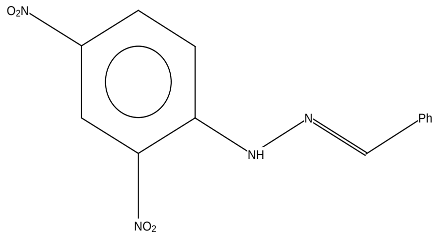

WUXHAY01

Reference: Srikala Pangannaya, D.R.Trivedi (2017) *Sens.Actuators,B* , **247**,673

Formula: $C_{13} H_{10} N_4 O_4$

Compound Name: (E)-1-benzylidene-2-(2,4-dinitrophenyl)hydrazine

Space Group:	P21/c	***Cell:***	***a*** 13.296(0)	***b*** 6.825(0)	***c*** 14.345(0)		
Space Group No.:	14	***(Å,°)***	α 90.00	β 92.56(0)	γ 90.00		
R-Factor (%):	4.05	***Temperature(K):***	296	***Density(g/cm³):***	1.462		

Parameters

Fragment 1
ANG1 (A) 3.328
DIST1 (D) 2.014

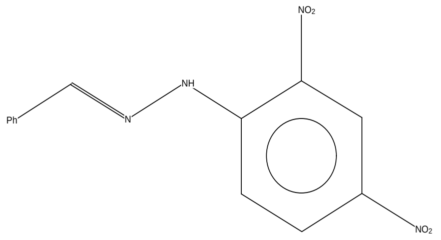

Search: search1 (Mon May 06 11:18:27 2019): Hits 65-68

XICKUQ

Reference: Gen-Rong Qiang, Zheng Fan, Shang Shan, Yu-Liang Tian, Xu-Chun Fu (2007) *Acta Crystallogr.,Sect.E:Struct.Rep.Online* ,**63,**o2246

Formula: $C_{14} H_{11} N_5 O_6$

Compound Name: (E)-4-Methylbenzaldehyde 2,4,6-trinitrophenylhydrazone

Space Group:	P-1	***Cell:***	***a*** 7.138(1)	***b*** 7.491(1)	***c*** 14.995(1)
Space Group No.:	2	***(Å,°)***	α 76.19(0)	β 79.47(0)	γ 83.61(0)

R-Factor (%): 6.60 ***Temperature(K):*** 294 ***Density(g/cm3):*** 1.502

Parameters

Fragment 1
ANG1 (A) 4.867
DIST1 (D) 4.428

Fragment 2
ANG1 (A) 4.867
DIST1 (D) 3.786

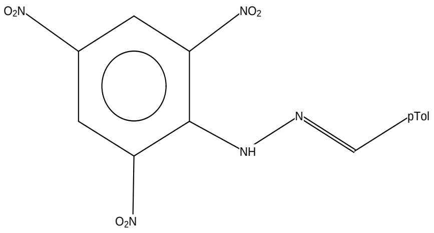

XOGWEX

Reference: K.Tiwari, M.Mishra, V.P.Singh (2014) *RSC Advances* ,**4,** 27556

Formula: $C_{13} H_{10} N_4 O_6,C_2 H_6 O_1 S_1$

Compound Name: 4-((2,4-dinitrophenyl)carbonohydrazonoyl)benzene-1,3-diol dimethyl sulfoxide solvate

Space Group:	P21/c	***Cell:***	***a*** 19.299	***b*** 6.620	***c*** 14.534
Space Group No.:	14	***(Å,°)***	α 90.00	β 102.77	γ 90.00

R-Factor (%): 6.94 ***Temperature(K):*** 293 ***Density(g/cm3):*** 1.454

Parameters

Fragment 1
ANG1 (A) 1.952
DIST1 (D) 1.958

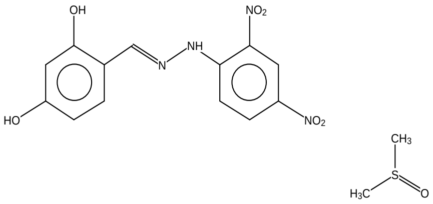

YEFCIV

Reference: C.J.Adams, M.I.Bruce, P.A.Duckworth, P.A.Humphrey, O.Kuhl, E.R.T.Tiekink, W.R.Cullen, P.Braunstein, S.C.Cea, B.W.Skelton, A.H.White (1994) *J.Organomet.Chem.* ,**467,**251

Formula: $C_{36} H_{19} N_4 O_{15} Os_3 P_1$

Compound Name: Undecacarbonyl-((2-(2,4-dinitrophenyl)methylhydrazonophenyl)-diphenylphosphine-P)-tri-osmium

Space Group:	P-1	***Cell:***	***a*** 15.909(6)	***b*** 15.511(5)	***c*** 9.187(3)
Space Group No.:	2	***(Å,°)***	α 87.89(3)	β 83.57(3)	γ 62.70(3)

R-Factor (%): 4.20 ***Temperature(K):*** 295 ***Density(g/cm3):*** 2.239

Parameters

Fragment 1
ANG1 (A) 7.008
DIST1 (D) 1.883

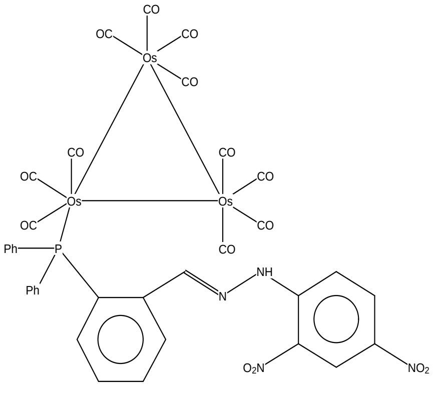

YEFFAR

Reference: Zuo-Liang Jing, Yu Liu, Xin Chen, Yu Ming (2006) *Acta Crystallogr.,Sect.E:Struct.Rep.Online* ,**62,**o862

Formula: $C_{14} H_{12} N_4 O_5$

Compound Name: N-(2,4-Dinitrophenyl)-N'-(4-methoxybenzylidene)hydrazine

Space Group:	P21/n	***Cell:***	***a*** 6.189(2)	***b*** 8.581(3)	***c*** 26.669(11)
Space Group No.:	14	***(Å,°)***	α 90.00	β 96.39(0)	γ 90.00

R-Factor (%): 3.35 ***Temperature(K):*** 294 ***Density(g/cm3):*** 1.493

Parameters

Fragment 1
ANG1 (A) 1.761
DIST1 (D) 3.834

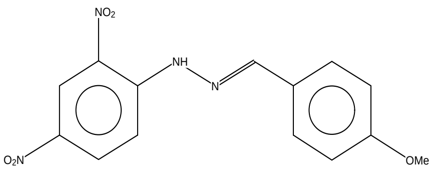

Search: search1 (Mon May 06 11:18:27 2019): Hits 69-71

YERWUO

Reference: Jun Shi (2006) *Acta Crystallogr.,Sect.E:Struct.Rep.Online* , **62,**o4533

Formula: $C_{20}\ H_{15}\ Cl_1\ N_4\ O_5$

Compound Name: (E)-1-(4-(4-Chlorobenzyloxy)benzylidene)-2-(2,4-dinitrophenyl) hydrazine

Space Group:	P-1	***Cell:***	***a*** 7.091(1)	***b*** 11.975(3)	***c*** 12.121(3)
Space Group No.:	2	***(Å,°)***	α 69.86(0)	β 78.46(0)	γ 85.19(0)

R-Factor (%): 4.04 ***Temperature(K):*** 294 ***Density(g/cm^3):*** 1.497

Parameters

Fragment 1
ANG1 (A) 5.354
DIST1 (D) 2.006

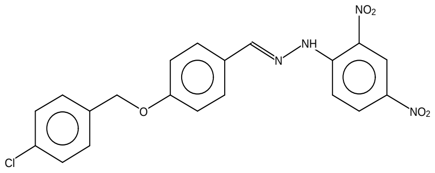

YIXQAY

Reference: A.A.Tameem, B.Saad, A.M.Salhin, S.R.Jebas, Hoong-Kun Fun (2008) *Acta Crystallogr.,Sect.E:Struct.Rep.Online* ,**64,** o679

Formula: $C_{13}\ H_9\ N_5\ O_7$

Compound Name: 2-Hydroxy-5-nitrobenzaldehyde 2,4-dinitrophenylhydrazone

Space Group:	P21/n	***Cell:***	***a*** 12.754(0)	***b*** 8.190(0)	***c*** 13.862(0)
Space Group No.:	14	***(Å,°)***	α 90.00	β 112.68(0)	γ 90.00

R-Factor (%): 5.03 ***Temperature(K):*** 100 ***Density(g/cm^3):*** 1.726

Parameters

Fragment 1
ANG1 (A) 4.629
DIST1 (D) 2.071

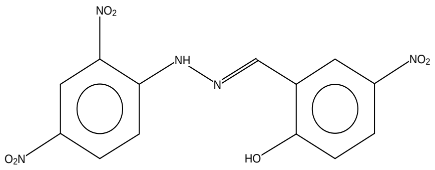

YIXQAY01

Reference: P.Ghosh, B.G.Roy, S.K.Mukhopadhyay, P.Banerjee (2015) *RSC Advances* ,**5,**27387

Formula: $C_{13}\ H_9\ N_5\ O_7$

Compound Name: 2-((2,4-dinitrophenyl)carbonohydrazonoyl)-4-nitrophenol

Space Group:	P21/n	***Cell:***	***a*** 12.745(5)	***b*** 8.223(5)	***c*** 13.910(5)
Space Group No.:	14	***(Å,°)***	α 90.00	β 112.26(0)	γ 90.00

R-Factor (%): 3.50 ***Temperature(K):*** 150 ***Density(g/cm^3):*** 1.710

Parameters

Fragment 1
ANG1 (A) 4.417
DIST1 (D) 3.783

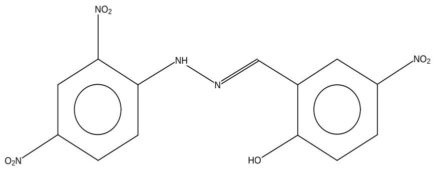

Supplement: Supplementary file 3 [file e-75-00770-sup3.pdf]
